# FTDZOA: An Efficient and Robust FS Method with Multi-Strategy Assistance

**DOI:** 10.3390/biomimetics9100632

**Published:** 2024-10-17

**Authors:** Fuqiang Chen, Shitong Ye, Lijuan Xu, Rongxiang Xie

**Affiliations:** 1 Department of Artificial Intelligence, Guangzhou Huashang College, Guangzhou 511300, China; 13984938024@163.com (F.C.); fyys600@163.com (S.Y.); d104544@163.com (L.X.); 2 State Key Laboratory of Public Big Data, Guizhou University, Guiyang 550025, China

**Keywords:** fractional order search strategy, triple mean point guidance strategy, differential strategy, zebra optimization algorithm, feature selection, 65K05

## Abstract

Feature selection (FS) is a pivotal technique in big data analytics, aimed at mitigating redundant information within datasets and optimizing computational resource utilization. This study introduces an enhanced zebra optimization algorithm (ZOA), termed FTDZOA, for superior feature dimensionality reduction. To address the challenges of ZOA, such as susceptibility to local optimal feature subsets, limited global search capabilities, and sluggish convergence when tackling FS problems, three strategies are integrated into the original ZOA to bolster its FS performance. Firstly, a fractional order search strategy is incorporated to preserve information from the preceding generations, thereby enhancing ZOA’s exploitation capabilities. Secondly, a triple mean point guidance strategy is introduced, amalgamating information from the global optimal point, a random point, and the current point to effectively augment ZOA’s exploration prowess. Lastly, the exploration capacity of ZOA is further elevated through the introduction of a differential strategy, which integrates information disparities among different individuals. Subsequently, the FTDZOA-based FS method was applied to solve 23 FS problems spanning low, medium, and high dimensions. A comparative analysis with nine advanced FS methods revealed that FTDZOA achieved higher classification accuracy on over 90% of the datasets and secured a winning rate exceeding 83% in terms of execution time. These findings confirm that FTDZOA is a reliable, high-performance, practical, and robust FS method.

## 1. Introduction

With the booming development of artificial intelligence technology, an increasing number of fields need to utilize big data technology to enhance performance. Examples include medical processing [1,2,3,4,5,6], digital media [7], and natural language processing [8].

Unfortunately, however, the original dataset often contains a great deal of redundant information, which reduces the interpretability of the data and results in a waste of computational resources. To reduce the loss of computational resources in the process of data computation and improve the interpretability of data, it is usually necessary to reduce the noise of redundant information in the dataset, which is a key issue in the field of big data technology [9]. If the original dataset contains N feature information, the denoising process requires searching a combination of subsets in $2^{N}$. However, searching for the optimal subset of combinations from many combinations consumes a power-exponential amount of computational cost [10]. To reduce the cost of the denoising process, utilizing the FS algorithm to cope with this NP-hard problem will appear more appropriate [11]. Therefore, the key research issue of this study aims to propose a reliable, robust, and practical feature selection method to reduce the loss of computational resources during data computation and enhance the interpretability of the data.

Currently, the main common FS algorithms are filter and wrapper methods [12]. Among them, the filter method evaluates the statistical properties or information-theoretic metrics between features and target variables and then cleans the redundant features by these information-theoretic metrics, which is advantageous in terms of high computational efficiency and small computational cost. However, the filter method cannot fully consider the characteristics of the subsequent learner, which leads to the poor performance of the selected feature subset on the learner, resulting in the lack of classification accuracy [13]. Unlike the filter approach, the wrapper approach treats the feature selection (FS) process as a search problem by continuously searching in the feature space and selecting features by evaluating the impact of different feature subsets on the model performance through learners such as K-Nearest Neighbors (KNN) [14], support vector machines [15], and neural networks [16], which in turn greatly ensures the classification accuracy. However, in wrapper methods, the feature subset search process using traditional methods will consume a lot of computational resources, while the meta-heuristic algorithm can effectively reduce the computational cost of the optimal combination search process due to its simplicity and flexibility [17].

Meta-heuristic algorithms are an optimization technique formed by simulating natural behavior, which has the advantages of easy implementation, high flexibility, and avoiding the trap of local optimality. They are currently categorized into four main types, which are evolutionary-based, chemical and physical-based, human-based, and population-based [18]. Among them, evolution-based algorithms commonly include the genetic algorithm [19], evolutionary strategies [20], and biogeography-based optimization [21]. Chemical and physical-based algorithms commonly include thermal exchange optimization [22] and the big bang–big crunch algorithm [23]. Human-based algorithms include teaching–learning-based optimization [24] and search and rescue optimization [25]. Population-based algorithms mainly include the slime mold algorithm [26], competitive swarm optimizer [27], and salp swarm algorithm [28].

Due to the simplicity and flexibility of the meta-heuristic algorithm, which makes it possible to effectively reduce the cost of the optimal feature subset search process, many scholars have proposed many wrapper methods based on the meta-heuristic algorithm to solve the FS problem. For example, Wang et al. proposed a binary grey wolf optimizer for solving the FS problem by combining foraging–following and Lévy flight strategies, called BFLGWO. By combining the stochastic nature of the learning strategy, the disadvantage of premature convergence was avoided, and the accuracy was improved to 4% on 12 datasets. Despite the improvement in accuracy, however, it suffered a substantial loss in time consumption, resulting in a less practical algorithm [29]. Mostafa et al. proposed an adaptive hybrid mutated differential evolution for feature dimensionality reduction in medical datasets, called A-HMDE, which, combined with the adaptive dynamic adjustability of the hybrid mutated strategy, enabled it to maintain a classification accuracy of more than 88% on different datasets, but its performance in removing redundant features is not obvious and there is still room for enhancement [30]. Gao et al. proposed a particle swarm algorithm based on information gain ratio-based sub-feature grouping to solve the FS problem, called ISPSO, due to the dynamically adjustable type of information gain ratio used, which makes it achieve good experimental results in eliminating the redundant features, and its disadvantage lies in the fact that the indexes considered are too singular, and no comprehensive consideration is given to the disadvantage, that the indexes considered are too single without comprehensive consideration of the indexes involved in FS [31]. Malik Braik et al. proposed an improved capuchin search algorithm to solve the FS problem by combining the chaotic strategy to address the problem that the capuchin search algorithm is prone to falling into the local optimum, called LCBCSA, and the proposed method has achieved good advantages in terms of accuracy and fitness value, but it suffers from the problem of falling into the local optimum when solving the high-dimensional FS problem, which is the problem of easily falling into local optimization, which makes its performance of high-dimensional FS suffer [32]. 

Heba Askr et al. proposed an improved golden jackal optimization algorithm in combination with copula entropy to solve the high-dimensional FS problem, called BEGJO, due to the dynamic adjustability of copula entropy during the search process, and it has achieved a great advantage in processing accuracy. However, for this reason, it sacrifices the feature dimension and running time, resulting in the reliability and practicality of the algorithm not being well guaranteed [33]. Mahmoud Abdel-Salam et al. proposed an adaptive chaotic dynamic learning gazelle optimization algorithm to solve the FS problem, called ACD-GOA, which, combined with adaptive inertia weights, improves the classification accuracy to more than 78%, but it still suffers from insufficient global search performance in solving the high-dimensional FS problem, and there is still the problem of insufficient global search performance, resulting in redundant features that cannot be effectively eliminated [34]. Law Kumar Singh et al. proposed a hybrid optimization algorithm by combining the emperor penguin optimization algorithm and bacterial foraging optimization algorithm to solve the FS problem, called EPO-BFO. Although it achieved good results in low-dimensional problems results, its accuracy is not guaranteed as the dimensionality increases [35]. For a deeper understanding, we summarize the advantages and disadvantages of the above work in terms of accuracy, execution time, error rate, and amount of feature dimensionality reduction in Table 1, where ‘Yes’ indicates that the indicator is assured and ‘No’ indicates that the indicator is not assured.

The above facts confirm that the FS methods based on meta-heuristic algorithms have strong feature dimensionality reduction performance, but although good progress has been made in classification accuracy, it has to be admitted that there is often a problem of falling into the local optimal feature subset in dealing with the high-dimensional FS problem, resulting in the loss of classification accuracy in high-dimensional datasets, which is due to the fact that the algorithm’s global optimal search performance has some. The essential reason is that the algorithm’s global optimization performance is somewhat insufficient. At the same time, with the gradual complexity of the data environment, the current FS algorithms have the inability to weigh indicators such as running time, feature subset size, and accuracy, thus limiting the effectiveness of capturing the intrinsic patterns and features of the data, resulting in the FS method not being guaranteed to be practical and reliable. The above facts motivate us to explore a novel and suitable meta-heuristic algorithm with efficient search performance to ensure that the algorithm can fully explore the search space when performing feature dimensionality reduction and to alleviate the problem of falling into a locally optimal subset of features due to high dimensional data. Fortunately, the zebra optimization algorithm (ZOA) has been shown to be a robust tool with efficient search capability [36]. Related studies have shown that ZOA has strong exploration capabilities and application scalability. For example, ZOA has been successfully applied to solve the transmission expansion planning problem [37], the renewable energy distributed energy system problem [38], and the cyber threat detection problem [39]. In addition, existing papers have not attempted to solve the FS problem with ZOA, and to fill this application gap, we apply ZOA to solve the FS problem. Meanwhile, considering that ZOA may still have the problem of falling into the local optimal feature subset when solving the high-dimensional FS problem, an improved ZOA is proposed to solve the FS problem in this paper by combining the fractional order search strategy, triple mean point guidance strategy, and differential strategy, and an improved ZOA is proposed to solve the FS problem, known as the FTDZOA.

By analyzing the status of the most novel FS methods mentioned above, we find that the problems of the current FS methods mainly focus on the following points. First, the algorithms excessively pursue classification accuracy but do not fully consider the significance of the execution time in the actual environment, which makes the proposed FS methods not guaranteed in terms of practicality. Secondly, some algorithms consider the indexes to be too homogenized and do not comprehensively consider the indexes involved in the FS problem, which makes the reliability of the algorithms lower. Meanwhile, most of the algorithms have excellent performance when dealing with low-dimensional FS problems, but there is the problem of easily falling into the local optimal subset on high-dimensional FS problems, which leads to a decrease in the algorithm’s solving ability. Compared with the existing FS methods, FTDZOA has its special features. Aiming at the problems of existing FS methods, firstly, the ability of the fractional order search strategy to retain information about the previous generation of individuals is fully utilized in order to reduce the execution time during the solution process and thus increase the utility of the algorithm. Secondly, this paper fully considers the classification accuracy, feature subset size, execution time, and error rate to comprehensively analyze and evaluate the FS problem. At the same time, the triple mean point guidance strategy and a differential strategy are used to enhance the global exploration capability of the algorithm by combining the information of the global optimal point, random point, and current point, as well as combining the information difference between different individuals to avoid the local optimization trap problem when FS methods solve high-dimensional problems. The above improvements make FTDZOA highly reliable as well as practical, and it can be considered a promising FS method. The main contributions of this paper are as follows:

- The fractional order search strategy is introduced to improve the exploitation of ZOA in solving FS problems.
- The introduction of the triple mean point guidance strategy effectively improves the exploration capability of ZOA and also ensures the exploitation of the algorithm.
- Introducing a differential strategy to enhance the global exploration capability of ZOA.
- A FS method based on FTDZOA is proposed by combining the above strategies.
- The FTDZOA-based FS method is used for 23 FS problems and achieves efficient performance.

The remainder of the paper is organized as follows: Section 2 focuses on the theoretical approach of ZOA. Section 3 proposes FTDZOA by introducing a fractional order search strategy, triple mean point guidance strategy, and differential strategy on the basis of ZOA. Section 4 applies the proposed FTDZOA-based FS method to solve 23 FS problems involving low, medium, and high dimensions to evaluate the FS performance of FTDZOA. Section 5 summarizes the conclusions of this paper and the future work schedule.

## 2. Zebra Optimization Algorithm

ZOA [36] is a novel optimization algorithm developed by modeling the behavior of zebra groups, which is mainly inspired by the zebra’s foraging behavior and defense strategies in the environment. ZOA mainly consists of an initialization phase, a foraging phase, and a defense phase during execution. In this section, we give the mathematical model of the above stages along with the complete execution of ZOA.

### 2.1. Initialization Phase

Like other metaheuristic algorithms, the execution of ZOA starts with initializing the population. In this section, the mathematical model of the initialization process of ZOA is presented. Each zebra individual corresponds to a solution of the problem to be solved, and by modeling each zebra individual using vectors, it is then possible to mathematically recognize the problem to be solved. At the same time, multiple zebra individuals are mathematically modeled to form an initialization population, which in turn starts the optimization iteration process. The initialization process is represented as Equation (1).

(1) $$X={\left[\begin{array}{c} X_{1}\\ \vdots \\ X_{i}\\ \vdots \\ X_{N} \end{array}\right]}_{N\times D}={\left[\begin{array}{ccccc} x_{1,1} & \cdots & x_{1,j} & \cdots & x_{1,D}\\ \vdots & \ddots & \vdots & \ddots & \vdots \\ x_{i,1} & \cdots & x_{i,j} & \cdots & x_{i,D}\\ \vdots & \ddots & \vdots & \ddots & \vdots \\ x_{N,1} & \cdots & x_{N,j} & \cdots & x_{N,D} \end{array}\right]}_{N\times D}$$

where X denotes the zebra population, $X_{i}$ denotes the ith zebra individual, N denotes the population size, D denotes the dimension of the problem to be solved, and $x_{i,j}$ denotes the value of the jth variable for the *ith* individual.

Each zebra represents a candidate solution to the problem to be solved, and the quality of the different individual zebras is usually evaluated using the fitness value, expressed using Equation (2).

(2) $$F={\left[\begin{array}{c} F_{1}\\ \vdots \\ F_{i}\\ \vdots \\ F_{N} \end{array}\right]}_{N\times 1}={\left[\begin{array}{c} F(X_{1})\\ \vdots \\ F(X_{i})\\ \vdots \\ F(X_{N}) \end{array}\right]}_{N\times 1}$$

where F denotes the vector of fitness values for the zebra population and $F_{i}$ denotes the fitness value of the ith individual. If the problem to be solved is a minimization optimization problem, the smaller the fitness value, the higher the quality of the individual and vice versa.

### 2.2. Foraging Phase

After initializing the population, individual positions are updated through the zebra’s foraging behavior in search of higher solution quality, and the mathematical model of the foraging phase is expressed as Equation (3).

(3) $$x_{i,j}^{new,P1}=x_{i,j}+r\cdot (PZ_{j}-I\cdot x_{i,j})$$

where $x_{i,j}^{new,P1}$ denotes the new value of the jth variable of the ith individual after passing through the foraging phase, r denotes a random number in the interval [0, 1], $PZ_{j}$ denotes the value of the jth variable of the optimal individual in the population, and I is any value in the set {1, 2}. Subsequently, the quality of the new and old states of the individual zebra was compared using the fitness values, which led to the retention of the individuals, expressed using Equation (4).

(4) $$X_{i}=\left\{\begin{array}{ll} X_{i}^{new,P1} & F_{i}^{new,P1}<F_{i}\\ X_{i} & else \end{array}\right.$$

where $X_{i}^{new,P1}$ denotes the new position of the ith individual after updating through the foraging phase, and $F_{i}^{new,P1}$ is the fitness value of the new state of the individual zebra formed after the individual passes through the foraging phase.

### 2.3. Defense Phase

After a zebra individual has made a mass gain through foraging behavior, it will subsequently use defense behavior to make a gain in individual mass. The mathematical model of the defense phase is represented as Equation (5).

(5) $$x_{i,j}^{new,P2}=\left\{\begin{array}{l} x_{i,j}+R\cdot (2r-1)\cdot (1-\frac{t}{T})\cdot x_{i,j}\;\;\;\;\;P_{s}\leq 0.5\\ x_{i,j}+r\cdot (AZ_{j}-I\cdot x_{i,j})\;\;\;\;\;\;\;\;\;\;\;\;\;\;\;\;\;\;\;\;else \end{array}\right.$$

where $x_{i,j}^{new,P2}$ denotes the new value of the jth variable of the ith individual after passing through the defense phase, R is a constant taking the value of 0.01, t denotes the current number of iterations of the algorithm, T denotes the maximum number of iterations of the algorithm, $P_{s}$ is a random number in the interval [0, 1], and $AZ_{j}$ denotes the value of the jth variable of a random zebra individual in the population. Subsequently, the quality of the new and old states of the individual zebra was compared using the fitness values, which led to the retention of the individuals, expressed using Equation (6).

(6) $$X_{i}=\left\{\begin{array}{ll} X_{i}^{new,P2} & F_{i}^{new,P2}<F_{i}\\ X_{i} & else \end{array}\right.$$

where $X_{i}^{new,P2}$ denotes the new position of the ith individual after updating through the defense phase, and $F_{i}^{new,P2}$ is the fitness value of the new state of the individual zebra formed after the individual passes through the defense phase.

### 2.4. Implementation of ZOA

**Figure 1:**
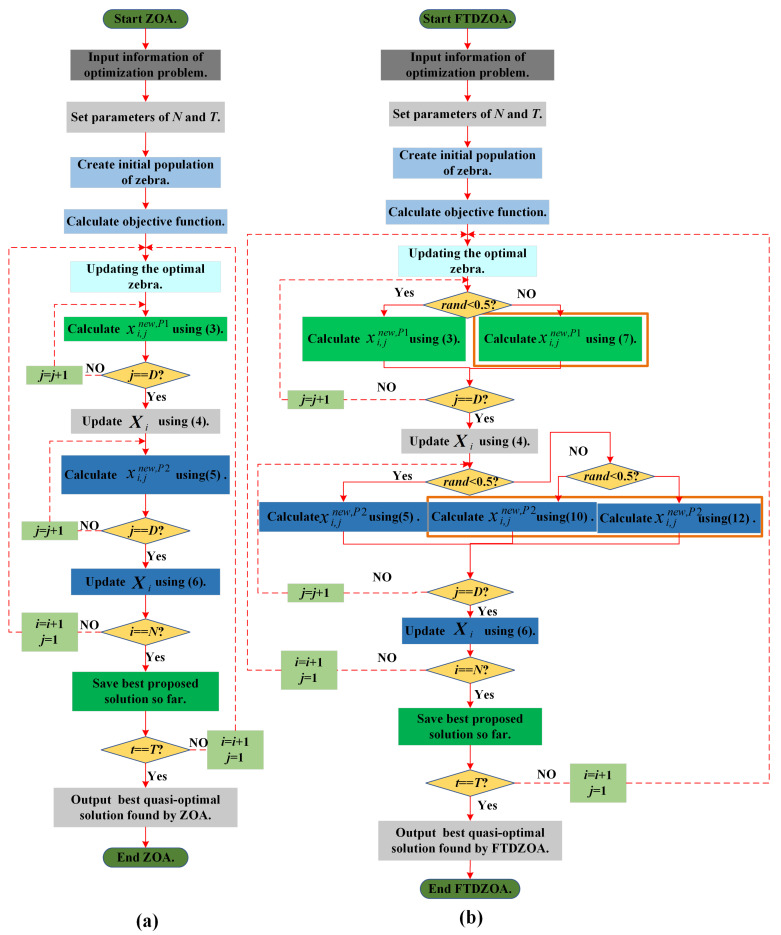
(**a**) The flowchart of ZOA algorithm. (**b**) The flowchart of FTDZOA algorithm.

This section focuses on the specific execution of the ZOA implementation process. At the end of the initialization population, the zebra individual positions are updated through the foraging phase and the defense phase, which in turn improves the quality of the solution until the optimal solution to the problem to be solved is output after the iteration stopping condition is reached. The flowchart of the algorithm is shown in Figure 1a.

## 3. Mathematical Modeling of FTDZOA

As the complexity of the current FS problem increases, solving the FS problem requires searching among an exponentially growing number of combinations, and the original ZOA is prone to falling into a locally optimal subset of features during the search process due to its insufficient exploration performance and exploitation performance, resulting in a lower classification accuracy and an increase in the running time. To improve the above drawbacks, this section proposes FTDZOA by combining three learning strategies. Firstly, the fractional order search strategy is introduced to enhance the exploitation ability of ZOA in solving the FS problem, which makes full use of the retention ability of the fractional order search strategy on the information of the previous generation of individuals and improves the classification accuracy. Secondly, the triple mean point guidance strategy is introduced, which combines the information of the global optimal point, random point, and current point to effectively enhance the exploration ability of ZOA, and ensures the exploitation ability of the algorithm so that the convergence performance of the algorithm is effectively enhanced. Finally, the exploration ability of ZOA is enhanced by introducing the differential strategy, which combines the information difference between different individuals and reduces the risk of falling into the subset of local optimal features. The introduction of the fractional order search strategy, triple mean point guidance strategy, and differential strategy based on ZOA makes the algorithm effectively avoid falling into the problem of locally optimal feature subsets when solving the FS problem so that the algorithm can effectively downsize redundant feature attributes in the data set and, at the same time, ensure the classification accuracy of the algorithm. The strategies proposed above are described in detail in the subsequent subsections.

### 3.1. Fractional Order Search Strategy

Due to the FS problem that requires searching for the optimal feature subset in a power exponential number of combinations, the original ZOA has insufficient exploitation performance, which can easily lead to the inability to quickly and effectively locate the optimal feature subset, reduce classification accuracy, and increase runtime. The above deficiencies motivate us to propose learning strategies with stronger exploitation capabilities. For example, in the high-dimensional datasets, Meadelon and Isolet, the algorithm needs to search for feature subsets among $2^{500}$ and $2^{617}$ feature combinations, which requires the algorithm to perform fast and efficient exploitation of the locally optimal region after locking it in order to enhance the classification accuracy and reduce the execution time. Additionally, the fractional order can describe the dynamic behavior of the system more finely. In our algorithm, the fractional order search strategy makes the search process able to transition more smoothly by introducing fractional order derivatives, avoiding the jump phenomenon that may occur in the traditional integer order search, which in turn enhances the ability to develop in the local region and can effectively improve the algorithm’s search efficiency and accuracy. Especially when dealing with high-dimensional datasets, this smooth transition property helps the algorithm to better adapt to the local characteristics of the data and enhance the exploitation ability. Meanwhile, in the literature [40], it is pointed out that the fractional order search strategy, due to its excellent ability to consider individual historical information, enhances the ability of individuals to summarize themselves by storing individual historical information, thereby effectively enhancing the algorithm’s exploitation capability. Based on the above inspiration, we introduce a fractional order search strategy to enhance the exploitation performance of ZOA, improve the algorithm’s ability to locate the optimal feature subset, and enhance the classification accuracy of the dataset, and we give an anomaly to the algorithm. We give an expression for the order fractional order search strategy as shown in Equation (7) [40].

(7) $$\begin{array}{c} x_{i,j}^{new,P1}=\frac{1}{1!}qx_{i,j}^{t}+\frac{1}{2!}q(1-q)x_{i,j}^{t-1}+\frac{1}{3!}q(1-q)(2-q)x_{i,j}^{t-2}\\ \;\;\;\;\;\;\;\;\;\;\;\;+\frac{1}{4!}q(1-q)(2-q)(3-q)x_{i,j}^{t-3}+l_{i,j}^{2}\times (x_{i,j}^{t}-x_{k,j}^{t}) \end{array}$$

where $x_{i,j}^{new,P1}$ denotes the new value of the jth variable value of the ith individual after updating by fractional order search strategy, “!” denotes the factorial operation, $x_{i,j}^{t}$ denotes the jth variable value of the ith individual in the current iteration, $x_{i,j}^{t-1}$ denotes the jth variable value of the ith individual in the previous generation, and $x_{k,j}^{t}$ denotes the jth variable value of a random individual in the population in the current iteration. $l_{i,j}$ is a random number obeying a standard normal distribution, denoted as $l_{i,j}∼N(0,1)$. q is the adaptive factor expressed as Equation (8).

(8) $$q=\frac{1}{\left(1+e^{L}\right)}\cdot cos(2\cdot \pi \cdot L)$$

where e represents the exponential operation, L represents the adaptive decreasing factor, expressed as Equation (9).

(9) $$L=(-1\cdot (\frac{t}{T})-2)\cdot r+1$$

In Equation (7), it can be found that the update of individuals mainly relies on the information of the first four generations of individuals. By integrating and summarizing the historical information of the first four generations of individuals, the quality of the solution is effectively enhanced. At the same time, combined with the adaptive factor q and utilizing the adaptability of the strategy, the exploitation ability of the enhanced algorithm is greatly promoted, enabling the algorithm to better locate the optimal feature subset. At the same time, the fractional order search strategy not only combines individual historical information but also ensures population diversity by learning from random individuals, reducing the risk of falling into local optimal feature subsets due to over-exploitation.

### 3.2. Triple Mean Point Guidance Strategy

**Figure 2:**
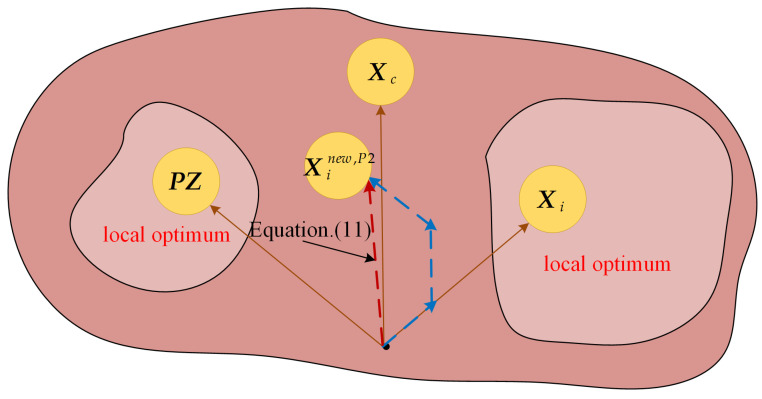
Triple mean point simulation diagram.

The original ZOA algorithm was designed to reduce redundant features in the dataset and improve the classification accuracy when solving FS problems. However, the number of combinations to be searched by the algorithm grows exponentially as the dimensionality of the data gradually increases. For example, in the datasets Clean and Semeion, the algorithm needs to search among $2^{167}$ and $2^{256}$ combinations of feature subsets, which makes the algorithm easily fall into the trap of locally optimal feature subsets due to the large search space and the limitations of the algorithm and makes it difficult to reduce the dimensionality of the dataset effectively. The root cause of the above problem is the insufficient global search performance of the algorithm. Therefore, the above facts inspire us to urgently propose a learning strategy with an efficient global search performance to improve the global search performance of the algorithm and increase the classification accuracy of the FS problem. Traditional mean point guidance methods tend to consider only a single mean point as the search direction, which may cause the algorithm to lack sufficient explorability in some cases. The literature [41] points out that the global search capability of the algorithm can be enhanced by generating new individuals by averaging the current individual with random individuals. Considering the high randomness of the individuals generated by averaging current individuals with random individuals, although it can effectively improve the global search ability of the algorithm, the exploitation ability cannot be guaranteed. Therefore, we consider the global optimal individual on this basis. The triple mean point guidance strategy in this paper provides more direction and diversity to the feature subset search process by introducing three different mean points. This strategy helps the algorithm to enhance its ability to explore unknown regions while maintaining convergence, thus exhibiting better performance when dealing with complex datasets with multiple local optima. The triple mean point guidance strategy is represented by Equation (10).

(10) $$x_{i,j}^{new,P2}=x_{i,j}\cdot r+(x_{m,j}+(1-\frac{t}{T})\cdot (x_{m,j}-x_{b,j}))\cdot (1-r)$$

where $x_{i,j}^{new,P2}$ represents the new value of the jth variable of the ith individual updated through the triple mean point guidance strategy, r represents the random number in the interval [0, 1], and $x_{b,j}$ represents the jth variable value of the random individual in the population. $x_{m,j}$ represents the jth variable value of the new individual generated through the triple mean point, expressed as Equation (11), and the simulation of triple mean point is shown in Figure 2.

(11) $$x_{m,j}=\frac{\left(x_{c,j}+PZ_{j}+x_{i,j}\right)}{3.0}$$

where $x_{c,j}$ represents the jth variable value of a random individual in the population, and $PZ_{j}$ represents the jth variable value of the globally optimal individual in the population.

From Figure 2, it can be seen that the triple mean point individuals help individuals escape from local optimal traps, enhance the algorithm’s exploration ability, and also have advantages in improving the accuracy of solutions. As can be seen from Equation (10), the individual learns from the triple-mean individual, which, due to the randomness and optimality of the triple-mean individual, allows the individual to explore a wider range of potential optimal regions, and, at the same time, the exploitation performance is guaranteed to enhance the ability to locate the optimal subset of feature regions during the FS problem-solving. In addition, the individual also learns from the gap between three-mean individuals and random individuals, which further enhances the exploration ability of the algorithm. It should be noted that the process of learning from the gap between three-mean individuals and random individuals is adaptive; namely, with the increase in the number of iterations, the degree of learning is gradually reduced, which not only ensures that there is a very good performance of global search in the early iteration period and that the optimal feature subset region can be well localized but also guarantees the exploitation performance, which makes the algorithm able to further exploit the potential optimal feature subset region and realize the improvement of classification accuracy.

### 3.3. Differential Strategy

**Figure 3:**
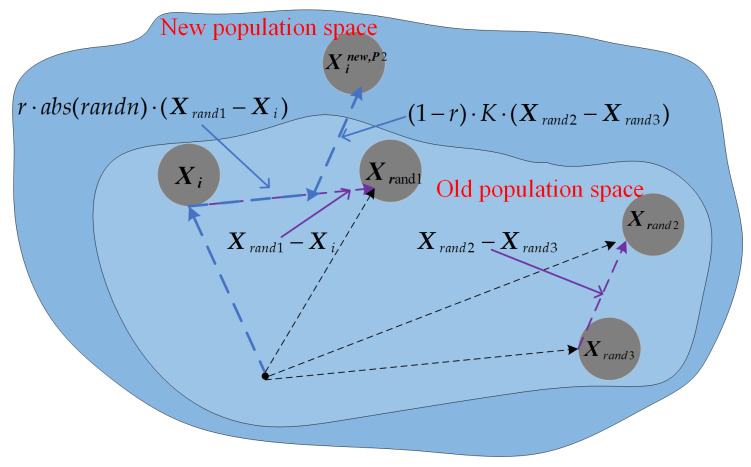
The differential strategy simulation diagram.

When solving FS problems, due to the diversity of the feature subset combinations, ZOA is prone to getting trapped in suboptimal feature subsets when searching for the optimal feature subset. The reason is that the algorithm has low population diversity during execution, which makes it easy to fall into the trap of suboptimal feature subsets, and it cannot effectively eliminate redundant features in the dataset. In the literature [42], it is pointed out that the differential strategy helps to enhance the population diversity of the algorithm and alleviate the problem of falling into local optimal traps. Therefore, in order to improve the performance of the algorithm in solving FS problems, this subsection proposes a novel differential strategy that combines traditional differential strategies to enhance population diversity during the algorithm execution, which is expressed as Equation (12), and the simulation is shown in Figure 3.

(12) $$\begin{array}{c} x_{i,j}^{new,P2}=x_{i,j}+r\cdot abs(randn)\cdot (x_{rand1,j}-x_{i,j})\\ +(1-r)\cdot K\cdot (x_{rand2,j}-x_{rand3,j}) \end{array}$$

where $x_{i,j}^{new,P2}$ represents the new value of the jth variable of the ith individual updated through differential strategy, while $x_{rand1,j}$, $x_{rand2,j}$, and $x_{rand3,j}$ represent the jth variable values of three random individuals in the population that are different from each other. The value of K is a random number in the set {0,1}, abs represents the absolute value operation, and randn denotes a random number that follows a normal distribution.

From Equation (12), it can be seen that the individual learns by learning from the gap between itself and a random individual and also learns from the gap between two random individuals and, by learning from different gaps, it enables the individual to receive more information and explore more promising optimal regions in the solution space, which greatly reduces the probability of falling into the trap of the local optimal subset. Meanwhile, in Figure 3, the blue dashed line indicates the generation process of the difference strategy, and it can be seen that with the individuals, after learning through the difference strategy, the population space becomes larger and the population diversity becomes higher, which is more promising to explore the optimal feature subset region. In summary, updating the position of individuals through the differential strategy makes the population diversity improve during the execution of the algorithm, which in turn can effectively downsize the redundant features in the dataset, reduce the running time, and improve the classification accuracy.

### 3.4. Implementation of FTDZOA

In view of the complexity of the current FS problem, ZOA is prone to falling into the subset of local optimal features during the search process due to the lack of its exploration performance and exploitation performance, which leads to the problem of not being able to effectively downsize the redundant features. In this section, FTDZOA is proposed by combining the fractional order search strategy, the triple mean point guidance strategy, and the differential strategy, so that the algorithm effectively avoids falling into the local optimal subset of features when solving the FS problem and makes the algorithm effectively downsize redundant feature attributes in the data set; at the same time, it ensures the classification accuracy of the algorithm. The implementation flowchart of the FTDZOA is shown in Figure 1b.

## 4. Results and Discussion

In this section, the performance of the FTDZOA-based FS method is mainly tested by using 23 datasets involving high-, medium-, and low-dimensional data for the experiments, followed by analyzing the population diversity, exploration–exploitation balance property, strategy effectiveness, fitness value, nonparametric test, convergence property, classification accuracy, feature subset size, running time, and comprehensive performance of FTDZOA. At the same time, the experimental performance comparison with nine efficient algorithms, namely ABO, DE, GWO, PSO, BOA, EO, MVO, WOA, and ZOA, objectively and fairly verifies that the FS method based on FTDZOA proposed in this paper has an efficient performance in solving FS problems. Among them, the parameter information of the nine comparison algorithms refers to the parameters set in the original literature of the corresponding algorithms in order to ensure the excellent performance of the algorithms. The information in the dataset used in this paper is shown in Table 2, and it can be found at the URL https://archive.ics.uci.edu/datasets (accessed on 15 October 2024). The details of the comparison algorithm parameter settings are shown in Table 3.

To ensure the fairness and reproducibility of the experiment, the experimental conditions were uniformly set to a population size of 10 and a maximum iteration count of 100. Each experiment was independently run 30 times without repetition to statistically analyze the experimental results. All experiments were conducted under hardware conditions of AMD Ryzen 6-core processor and 8Gb memory produced by Advanced Micro Devices (AMD) in California, USA. All code involved in the experiments was run in MATLAB R2021b on the Windows 11 system environment. Some of the codes involved can be found at the URL https://github.com/JingweiToo/Wrapper-Feature-Selection-Toolbox (accessed on 15 October 2024).

### 4.1. FS Optimization Model

The main purpose of the FS problem is to extract the effective feature information from the original chaotic data set and eliminate the redundant features, which in turn reduces the data complexity and improves the classification accuracy, and it can be regarded as a high-dimensional combinatorial optimization problem whose fitness function is defined as shown in Equation (13).

(13) $$min\;f(X_{i})=\lambda_{1}\cdot error+\lambda_{2}\cdot R/n$$

where $X_{i}$ denotes the ith solution, which is the valid subset of features extracted from the original dataset; error denotes the error rate of classification using this subset of features; R denotes the size of the proposed subset of features; and n denotes the size of the features in the original dataset. $\lambda_{1}\in [0,1]$, $\lambda_{2}=1-\lambda_{1}$. In this paper $\lambda_{1}$ takes the value 0.9.

**Figure 4:**
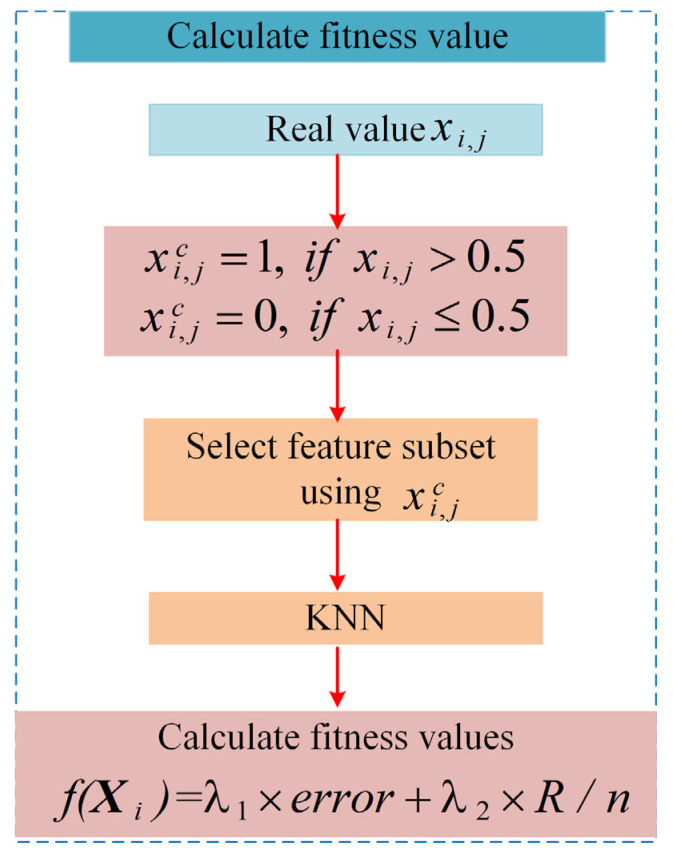
Flowchart for calculating the fitness value.

Since the individual information is always real-valued during the iterative process of FTDZOA, but due to the discrete type of the FS problem, it is necessary to convert the real-valued value to discrete-valued operation for the individual before calculating the fitness value, and the specific flowchart for calculating the fitness value is shown in Figure 4, with the main steps as follows:

**Step 1:** real-valued individual $X_{i}=(x_{i,1}\ldots x_{i,j}\ldots x_{i,D})$ is converted to a discrete-valued individual $X_{i}^{c}=(x_{i,1}^{c}\ldots x_{i,j}^{c}\ldots x_{i,D}^{c})$, expressed as the following Equation (14):

(14) $$x_{i,j}^{c}=\left\{\begin{array}{l} 1,x_{i,j}>0.5\\ 0,x_{i,j}\leq 0.5 \end{array}\right.\;i=1,2,...,N;j=1,2,...,D$$

**Step 2:** Selection of feature subsets in the original dataset by discrete-valued individual $X_{i}^{c}$, where $x_{i,j}^{c}=1$ means that the jth feature is selected and vice versa means that the jth feature is not selected.
**Step 3:** The selected subset of features is used to calculate the classification accuracy using KNN. In this paper, K takes the value of 5.
**Step 4:** Calculate the fitness value using Equation (13).

### 4.2. Population Diversity Analysis

**Figure 5:**
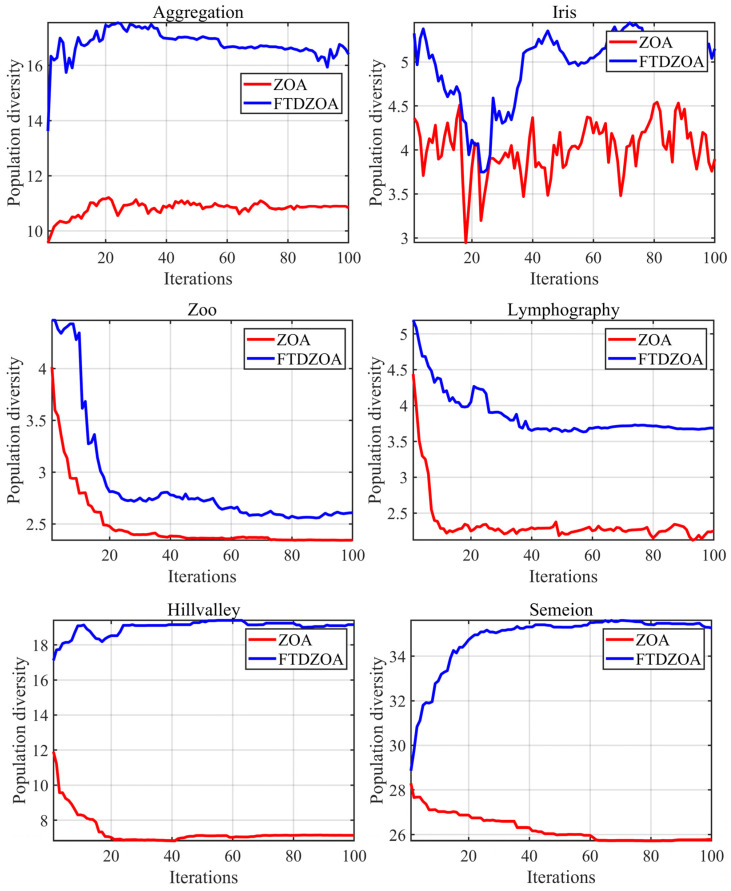
Population diversity on FS datasets.

In this section, we mainly analyze the population diversity of the FS method based on FTDZOA in solving the FS problem, and the higher population diversity indirectly reflects the algorithm’s ability to jump out of the locally optimal feature subset, which means that the algorithm is able to explore a wider subset of the optimal feature region and effectively eliminate the redundant features from the original dataset. The experimental results are shown in Figure 5, where the X-axis represents the number of iterations and the Y-axis represents the value of population diversity.

From Figure 5, it can be seen that the FTDZOA-based FS method has a higher population diversity than ZOA throughout the iteration process when performing data dimensionality reduction on the low-dimensional datasets Aggregation and Iris, which implies that the FTDZOA-based FS method possesses a stronger capability of jumping out of the locally optimal features subset when solving the low-dimensional FS problem. This is mainly due to the introduction of the triple mean point guidance strategy, and differential strategy in this paper, which improves the population diversity of the algorithm. Meanwhile, when dealing with the medium-dimensional FS problem, the population diversity of FTDZOA is higher than that of ZOA throughout the iterative process, which indicates that with the increase of data dimension, the triple mean point guidance strategy, and differential strategy proposed in this paper can still effectively enhance the algorithm’s population diversity and improve its ability to jump out of the local optimal feature subset. Finally, when dealing with high-dimensional FS problems, the algorithm needs a higher ability to jump out of the local optima due to the challenge of the complex search space brought by the dimension enhancement. Fortunately, FTDZOA effectively enhances the population diversity, and the population diversity is always ahead of ZOA when dealing with high-dimensional FS problems, which demonstrates that for high-dimensional FS problems, the triple mean point guidance strategy, and differential strategy proposed in this paper can still effectively and robustly enhance the population diversity and improve the algorithm’s performance in the high-dimensional FS problem. In summary, we can conclude that the triple mean point guidance strategy, and differential strategy proposed in this paper can effectively enhance the population diversity, improve the ability of the algorithm to jump out of the suboptimal feature subset, and effectively eliminate redundant features, regardless of the low-dimensional, medium-dimensional, and high-dimensional FS datasets.

### 4.3. Exploration-Exploitation Balance Analysis

**Figure 6:**
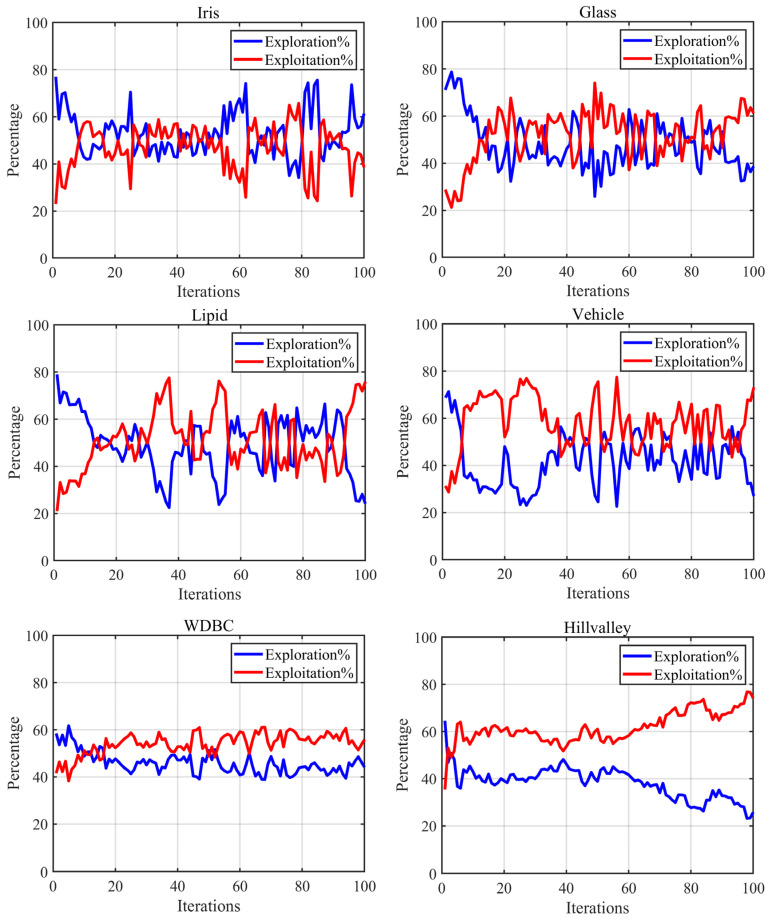
Exploration–exploitation ratio on FS datasets.

In this section, we mainly analyze the exploration–exploitation phase of the FTDZOA-based FS method when dealing with FS problems, in which the main purpose of the exploration phase is to explore the optimal region in the vast solution space, while the main purpose of the exploitation phase is to carry out a deeper step in the discovered optimal region to speed up the convergence speed and convergence accuracy. A good algorithm should achieve a good balance between these two phases in order to make the exploration and exploitation phases complement each other and jointly promote the performance of the algorithm. The experimental results are shown in Figure 6, where the X-axis represents the number of iterations and the Y-axis represents the proportion of exploration/exploitation phases in the running process.

From Figure 6, it can be seen that the FS method based on FTDZOA has a higher exploration ratio in the early stage of the iteration when solving the low-dimensional FS problem, which indicates that the algorithm has a higher global search capability in the early stage of the iteration when solving the low-dimensional FS problem, mainly due to the fact that the triple mean point guidance strategy and the differential strategy are introduced in this paper, which makes the exploration ability improved and enables the algorithm to locate the global optimal region more effectively. Subsequently, the exploitation ratio of the algorithm gradually increases, which is mainly due to the introduction of a fractional order search strategy in this paper, which improves the exploitation ability of the algorithm, and the increase of the exploitation ratio helps to further develop the optimal region and improve the classification accuracy. When solving the medium- and high-dimensional FS problem in the early iteration period, due to the introduction of triple mean point guidance strategy, and differential strategy, the algorithm has a stronger exploration performance and is able to locate the optimal region quickly and efficiently. Then, due to the introduction of a fractional order search strategy that is introduced, the exploitation phase is dominated and the algorithm’s local search capability is strengthened, which in turn improves the convergence performance of the algorithm and enables the algorithm to effectively eliminate redundant features. The above facts illustrate that due to the introduction of the three strategies in this paper, the performance of the FTDZOA-based FS method is enhanced when dealing with low, medium, and high-dimensional FS problems.

### 4.4. Strategies Effectiveness Testing

**Figure 7:**
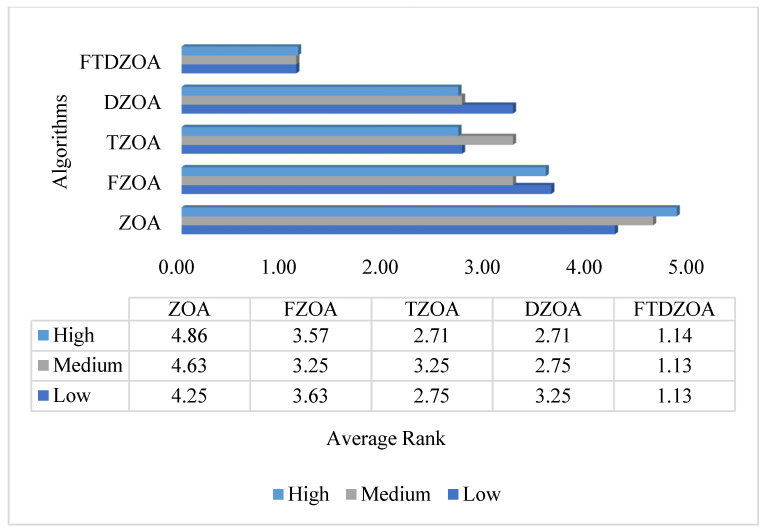
Strategies effectiveness evaluation bar chart.

Population diversity and exploration–exploitation capabilities during FTDZOA execution have been analyzed in the above subsections, but we have no way of knowing the improvement in algorithm performance for a single strategy. In this section, the effectiveness of the strategies is mainly evaluated: firstly, the fractional order search strategy is introduced into ZOA to form the algorithm FZOA, the triple mean point guidance strategy is introduced into ZOA to form the algorithm TZOA, and the differential strategy is introduced into ZOA to form algorithm DZOA. At the same time, the above three strategies are introduced into ZOA to form the algorithm FTDZOA. Subsequently, the FS performance of ZOA, FZOA, TZOA, DZOA, and FTDZOA is evaluated on 23 FS datasets, involving high dimensionality, medium dimensionality, and low dimensionality. The experimental results are shown in Figure 7, which shows the average ranking of the algorithm based on the fitness values on different dimensional datasets. From the figure, it can be seen that the introduction of the fractional order search strategy in ZOA avoids the jumping phenomenon that may occur in the traditional integer order search, and at the same time utilizes the individual history information and enhances the exploitation ability in the local area, which makes the average ranking of FZOA better than that of the original ZOA in different dimensional datasets. Meanwhile, the introduction of the triple mean point guidance strategy in ZOA avoids the limitation of the traditional mean point and strengthens the ability of the algorithm to jump out of the local optimal trap by combining the randomness and optimality of the strategy, which makes the average rankings of TZOA outperform those of the original ZOA on different dimensional datasets. The introduction of a differential strategy in ZOA improves the global search performance of the algorithm and enriches the optimal solution region of the problem due to the learning of individuals by learning from the differences in information from other individuals, which makes the average ranking of DZOA better than that of the original ZOA in different dimensional datasets. We can notice that the exploitation performance as well as the global search performance of FTDZOA is improved after the introduction of the above three strategies simultaneously in ZOA, and the average rankings on different dimensional datasets are optimal. Through the above analysis, we can conclude that the introduction of a fractional order search strategy, triple mean point guidance strategy, and differential strategy in ZOA alone can improve the performance of ZOA from different angles, which confirms that the proposed strategies in this paper are all effective. Meanwhile, we can find that the performance of ZOA can be improved by introducing the above three strategies at the same time.

### 4.5. Fitness Value Analysis

In this section, the main focus is to analyze the fitness value of the FTDZOA-based FS method in executing the FS problem, and at the same time, combine it with the comparison algorithm to provide an objective and fair assessment of the performance of the proposed method in the FS problem in this paper. In order to avoid the chance of experimental results, each group of experiments is executed independently and unrepeatedly for 30 times to count the experimental results. The experimental results are shown in Table 3, where “Best” denotes the optimal fitness value, “Mean” denotes the mean fitness value, “Worst” denotes the worst fitness value, “Rank” denotes the individual ranking on different metrics. “Mean Rank” denotes the mean ranking on different metrics and “Final Rank” denotes the final ranking obtained based on the “Mean Rank”. Among them, the optimal values on the corresponding indicators are represented in bold, and this is also the case in the following text.

**Figure 8:**
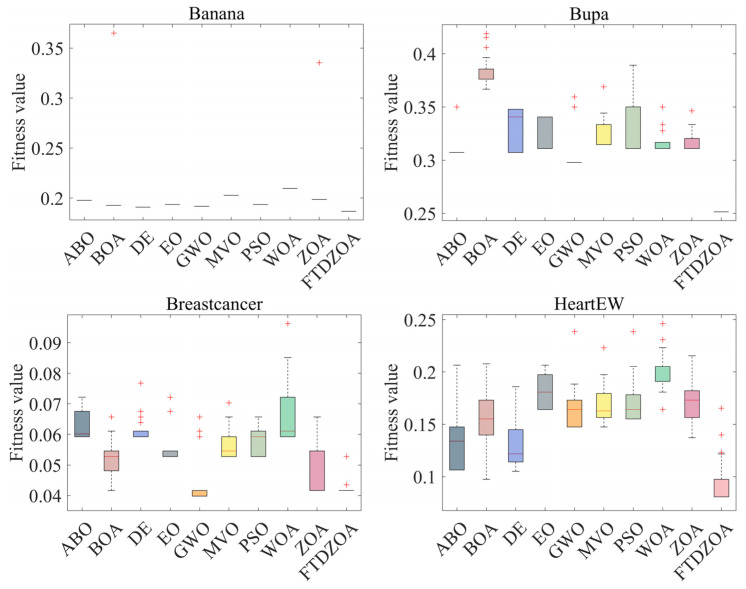
Box plots on low-dimensional FS problems.

From Table 4, it can be seen that the FS method based on FTDZOA solves the low-dimensional FS problem. In terms of the best fitness value, FTDZOA is ranked first with 88% probability, while ZOA is ranked first only with 25% probability, and at the same time, the winning rate of FTDZOA is ahead of the comparison algorithms. This shows that due to the introduction of the fractional order search strategy, the triple mean point guidance strategy, and differential strategy in this paper, the ability of the algorithm to jump out of the locally optimal subset of features is effectively improved, and the redundant features are effectively eliminated, which ensures the performance of the algorithm in the low-dimensional FS problem. However, it is undeniable that although FTDZOA achieves good results in the low-dimensional FS problem, it is weaker than GWO in the Breastcancer dataset, which indicates that the performance of FTDZOA proposed in this paper is still to be improved in some specific low-dimensional datasets. In terms of the mean fitness value, FTDZOA ranks first with a probability of 100%, ahead of ZOA as well as other comparison algorithms, which indicates that FTDZOA possesses high solution stability when dealing with the low-dimensional FS problem. Meanwhile, Figure 8 demonstrates the box plot of FTDZOA when dealing with the low-dimensional FS problem, from which it can be visualized that the box corresponding to FTDZOA has the smallest height, which indicates that FTDZOA possesses high solution stability in solving the low-dimensional FS problem and can be considered a robust FS method. In terms of the worst fitness value, FTDZOA ranks first with 100% probability, ahead of ZOA and other comparative methods, which indicates that the FS method based on FTDZOA possesses higher fault tolerance when solving low-dimensional FS problems, indirectly reflecting that FTDZOA is a reliable FS method. Meanwhile, compared to FTDZOA, the comparison algorithm cannot precisely locate the globally optimal region due to the insufficiency of the algorithm’s exploitation strategy when solving the low-dimensional FS problem, which makes the algorithm limited to the size of the search space, and it is unable to exploit the region efficiently, which leads to the algorithm not being able to fully explore all the possible combinations of features and thus not being able to find the optimal subset of features, resulting in the insufficient performance of the algorithm. Through the above analysis, it can be concluded that due to the fractional order search strategy, the triple mean point guidance strategy, and differential strategy introduced in this paper, the optimization performance, solution stability, and reliability of FTDZOA are improved. 

**Figure 9:**
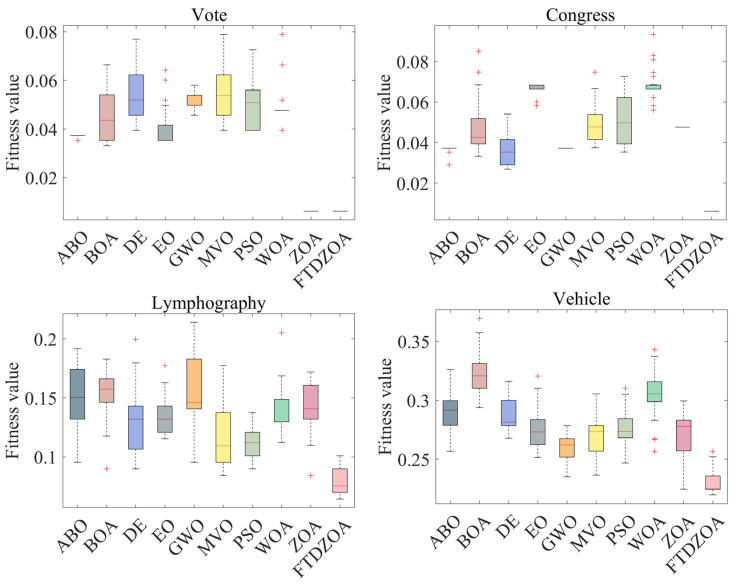
Box plots on medium-dimensional FS problems.

Secondly, it can be seen from Table 4 that the FS method is based on FTDZOA in solving the medium dimension FS problem. In terms of the best fitness value, FTDZOA ranks first with 100% probability while ZOA only ranks first with 13% probability, and at the same time, the winning rate of FTDZOA is ahead of the comparison algorithms. This demonstrates that as the dimensionality of the FS problem increases, the fractional order search strategy, triple mean point guidance strategy, and differential strategy proposed in this paper are still able to contribute to the performance of the algorithms in dealing with the FS problem, allowing the algorithms remain efficient in solving medium dimensional feature problems. In the mean fitness value, FTDZOA still ranks first with 100% probability, far ahead of ZOA and the other comparison algorithms, which shows that with the enhancement of the dimensionality and complexity of the FS problem, the FTDZOA proposed in this paper still possesses a high solution stability, which indirectly reflects that with the enhancement of the problem dimensions, the practicability of the FTDZOA has been more emphasized. At the same time, Figure 9 shows the box diagram of FTDZOA when dealing with the medium-dimensional FS problem, from which it can be intuitively seen that the corresponding box height of FTDZOA is the smallest, which indicates that the distribution of the solutions of FTDZOA is more clustered when solving the medium-dimensional FS problem, and this also reflects that in the realistic feature dimensionality reduction environment, the stability of the solution of FTDZOA is very high and the reliability is guaranteed, and it can be be considered a reliable FS method. In terms of the worst fitness value, FTDZOA ranks first with a probability of 88%, ahead of ZOA and other comparative methods, which indicates that with the improvement of the problem dimensions, the FS method based on FTDZOA also has a higher fault tolerance rate and also confirms its reliability in solving the medium-dimensional FS problem. Meanwhile, compared to FTDZOA, the comparison algorithm is limited by the search efficiency of the algorithm when solving the medium-dimensional FS problem. With the increase in the number of features, the complexity of the search space increases, and the algorithm needs to find the optimal solution in a larger solution space, so it needs a stronger ability to jump out of the trap of local optimality while the comparison algorithm, due to the limitations of the strategy, leads to the algorithm being prone to falling into the local optimality when solving the FS problem, and it is not able to carry out an effective spatial exploration in the large-scale combinations, resulting in a loss of performance. Through the above analysis, it can be concluded that due to the fractional order search strategy, triple mean point guidance strategy, and differential strategy introduced in this paper, FTDZOA can still effectively promote its performance in the face of the increase in the dimensionality of the FS problem. The performance of FTDZOA can still be effectively promoted and the reliability and robustness of the algorithm can be guaranteed.

**Figure 10:**
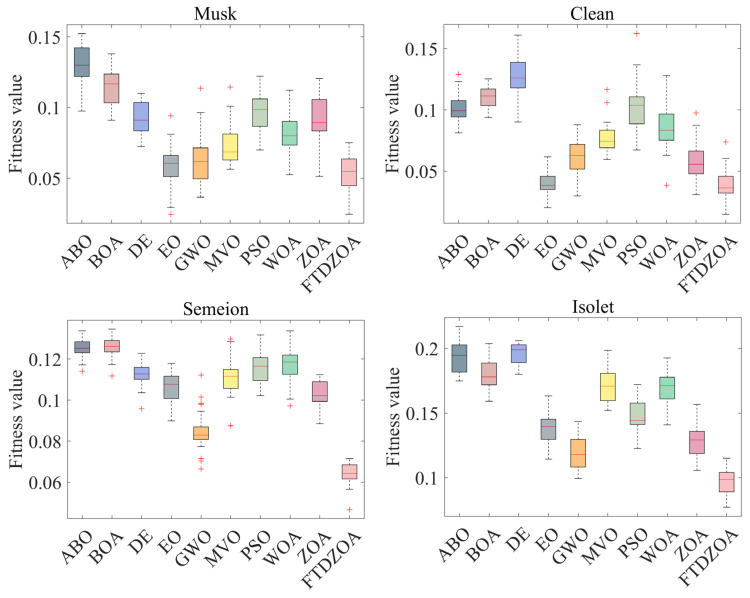
Box plots of high-dimensional FS problems.

From Table 4, it can be seen that the FTDZOA-based FS method ranks first with 100% probability in the best fitness value index when dealing with the high-dimensional FS problem, ahead of the other comparative algorithms, which confirms that the strategy introduced in this paper is still effective in dealing with the high-dimensional combinatorial optimization problem. Meanwhile, in the mean fitness value index, it ranks first with 100% probability, which demonstrates high stability in the high-dimensional FS problem. Figure 10 demonstrates the box plot of the FTDZOA-based FS in solving the high-dimensional FS problem, from which it can be seen that the height of the box of FTDZOA is the smallest in different high-dimensional datasets, which reflects that FTDZOA has a much better performance in the high-dimensional FS problems with higher solution stability. In the worst fitness metric, it ranks first with 86% probability and is considered to possess the highest fault tolerance. However, compared to FTDZOA, in high-dimensional FS problems, the comparison algorithm is limited by the dimensionality catastrophe, and the dimensionality of the search space increases dramatically as the number of features increases dramatically, while the global search capability of the comparison algorithm is limited by the search strategy, resulting in an extreme lack of population diversity, which leads to a sharp decrease in the search efficiency of the algorithm, which makes the algorithm’s FS performance drop drastically. In summary, the FTDZOA proposed in this paper has higher stability and reliability in high-dimensional FS problems due to the advanced nature of its strategy.

**Figure 11:**
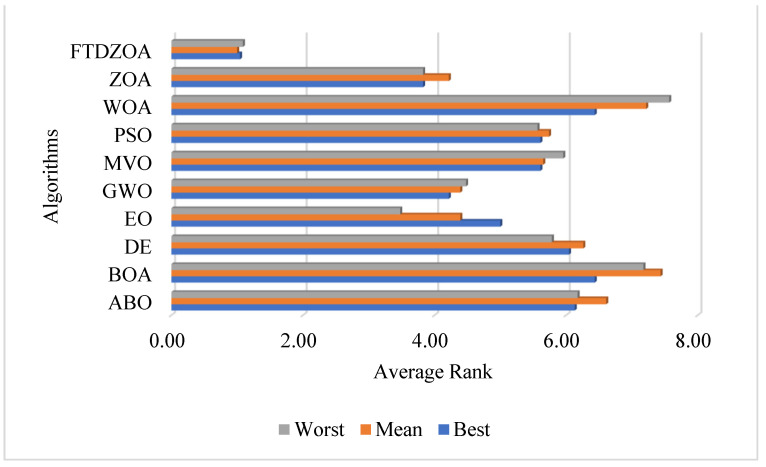
Ranking statistics chart based on fitness values.

Finally, from a comprehensive point of view, the FS method based on FTDZOA has an average ranking of 1.043 in the best fitness value metric in 23 FS problems involving low, medium, and high dimensions, ahead of the other comparative algorithms, reflecting that FTDZOA possesses stronger global optimization-seeking capability. In the mean fitness value index, the mean ranking is 1.000, which confirms that FTDZOA possesses a stronger solution stability. In addition, in the worst fitness value index, the average ranking is 1.087, which confirms that FTDZOA has a higher fault tolerance for solving. In order to see more intuitively the performance differences of different algorithms in each metric, Figure 11 shows the ranked histograms of the algorithms, from which it is intuitively clear that FTDZOA ranks not only ahead of ZOA in terms of the worst fitness, the mean fitness, and the best fitness values, but also ahead of the other excellent algorithms. In summary, we can conclude that the FS method based on FTDZOA is an efficient, robust, and reliable FS method.

### 4.6. Nonparametric Analysis

In some cases, relying solely on numerical results may not be sufficient to comprehensively and accurately assess the differences between the performance of different algorithms. This is because the numerical results are sensitive to outliers, and one or a few outliers may significantly change the numerical statistics, thus affecting the interpretation of the overall data. Therefore, in order to avoid performance bias due to outliers, in this section, we have performed a Friedman mean test with a significance factor of 0.05 for the experimental results to objectively and fairly assess the performance of the FTDZOA-based FS method. The experimental results are shown in Table 5, where “Mean Rank” denotes the average ranking of the algorithm on all datasets and “Final Rank” denotes the final ranking based on the “Mean Rank”. In addition to this, we also performed a Wilcoxon statistical rank sum test with a significance factor of 0.05 for the experimental results. The experimental results are shown in Table 6, where ‘+’ indicates that the algorithm performance is significantly better than FTDZOA, ‘−’ indicates that the algorithm performance is significantly weaker than FTDZOA, and ‘=’ indicates that the algorithm performance is not significantly different from FTDZOA.

As can be seen from Table 5, in the eight low-dimensional FS problems, the average ranking of the FTDZOA-based FS method through the Friedman mean test is 1.74, which is ahead of the other comparison algorithms. Meanwhile, the Friedman mean test ranking shows that the winning rate of FTDZOA in the low-dimensional FS problems is 100%, and the above results confirm the efficiency of the FTDZOA-based FS method in the low-dimensional FS problems. In the eight medium-dimensional FS problems, the average ranking of the FTDZOA-based FS method through the Friedman mean test is 1.4, which is ahead of the other comparative algorithms. Meanwhile, the winning rate of FTDZOA in the medium-dimensional FS problems is 100%. This demonstrates that as the dimensionality of the FS problem increases, the performance of FTDZOA proposed in this paper can still perform efficiently in finding the optimal performance. In the seven high-dimensional FS problems, the average ranking of the FS method based on FTDZOA through the Friedman mean test is 1.68, which is ahead of the other compared algorithms, and at the same time, the winning rate of FTDZOA in the seven high-dimensional FS problems is 100%, which confirms that the FTDZOA proposed in this paper still performs efficiently in solving high-dimensional FS problems. Meanwhile, from a comprehensive point of view, the average ranking of FTDZOA in 23 FS problems is 1.54, which is ahead of the other compared algorithms. Through the above analysis, we can conclude that the algorithm’s exploration and exploitation capabilities have been improved due to the introduction of the fractional order search strategy, triple mean point guidance strategy, and differential strategy, which makes the FTDZOA efficient in solving low, medium and high-dimensional FS problems, and it also confirms that FTDZOA is a robust and reliable FS method.

From Table 6, it can be seen that ABO, BOA, EO, GWO, and MVO perform significantly weaker than FTDZOA in low-dimensional datasets with 87.5% probability; DE, PSO, and WOA with 100% probability; and ZOA with 75% probability. The above statistical analysis of the results shows that due to the FTDZOA’s superiority in strategy, it makes its FS performance better than other algorithms in low-dimensional datasets. Meanwhile, ABO, BOA, DE, GWO, PSO, and WOA are weaker in significance than FTDZOA at 100% probability in the medium dimensional dataset. EO, MVO, and ZOA are significantly weaker than FTDZOA with 87.5% probability. The above analysis shows that the global search performance of FTDZOA is improved due to the introduction of the triple mean point guidance strategy as well as the differential strategy, which in turn enables it to explore the more promising optimal solution regions, which makes FTDZOA outperform the comparison algorithms on the medium-dimensional FS dataset. FTDZOA significantly outperforms ABO, BOA, DE, MVO, PSO, WOA, and ZOA in high dimensional datasets and achieves excellent results, which is mainly due to the fact that the strategy proposed in this paper improves the algorithm’s exploitation ability and global search ability and also confirms that the FS method based on FTDZOA proposed in this paper possesses powerful solving performance of the FS method.

### 4.7. Convergence Analysis

**Figure 12:**
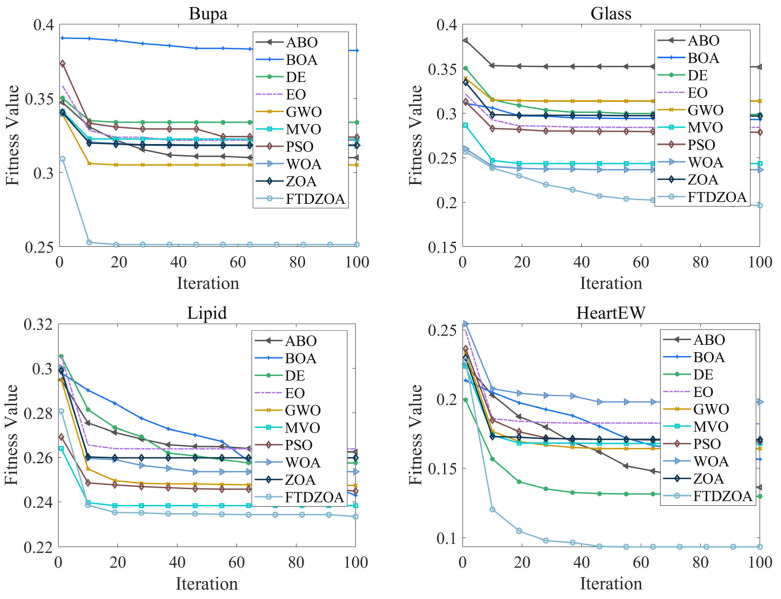
Convergence curves in low-dimensional FS problems.

**Figure 13:**
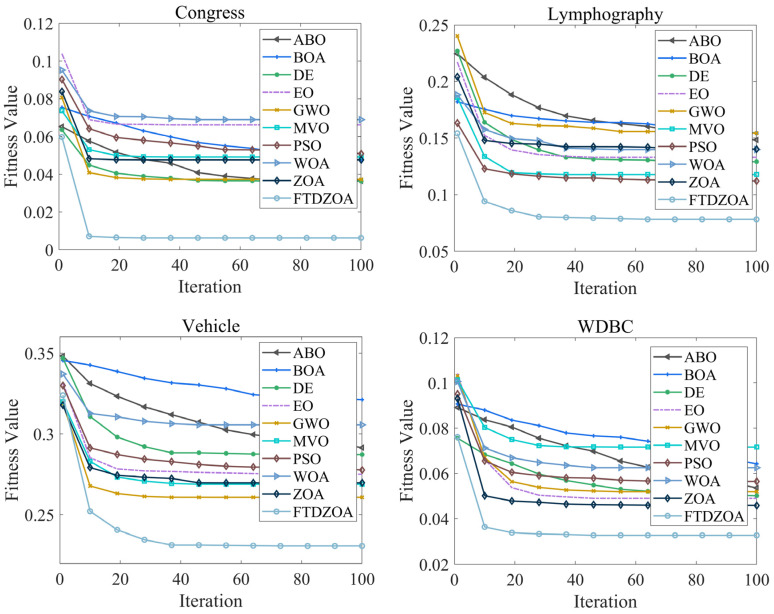
Convergence curves in the medium-dimensional FS problem.

**Figure 14:**
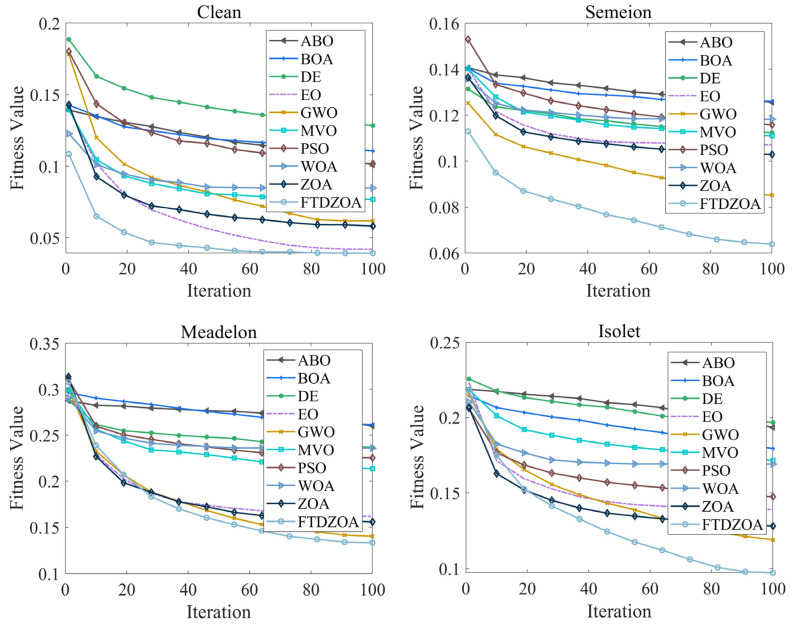
Convergence curves in the high-dimensional FS problem.

In the above subsection, the FS performance of the FTDZOA-based FS method is analyzed from the perspectives of population diversity, exploration and exploitation stage, fitness value, and stability, which confirms that FTDZOA possesses an efficient solution accuracy in solving the FS problem, and it can efficiently perform elimination of the redundant features and improve the classification accuracy. However, in addition to this, the convergence property of the algorithm is also important, which is directly related to the practicality and reliability of the algorithm. Figure 12 shows the convergence graph of FTDZOA in solving the low-dimensional FS problem, Figure 13 shows the convergence graph of FTDZOA in solving the medium-dimensional FS problem, and Figure 14 shows the convergence graph of FTDZOA in solving the high-dimensional FS problem, where the X-axis denotes the number of iterations and the Y-axis denotes the adaptation value.

It can be seen in Figure 12 that all the algorithms can effectively reduce the fitness value when solving the low-dimensional FS problem, and it is worth noting that in the 10th iteration, the FTDZOA has taken a clear convergence advantage in convergence accuracy, possessing faster convergence speed, which is mainly due to this paper’s fractional order search strategy, which enhances the local development ability of the algorithm, improves the convergence speed, and enhances the practicality of the algorithm. After the 10th iteration, FTDZOA still optimizes the FS problem steadily, which is mainly due to the introduction of the triple mean point guidance strategy, and differential strategy, which strengthen the global search ability of FTDZOA and further illustrates FTDZOA’s reliability. Through the above analysis, it is confirmed that when solving the low-dimensional FS problem, FTDZOA has the advantage of convergence performance due to the introduction of the learning strategy, which makes it more practical and reliable.

As can be seen from Figure 13, with the increase in the dimensionality of the FS problem, all algorithms are able to effectively reduce the fitness value when solving the medium-dimensional FS problem, and similarly, the introduction of the fractional order search strategy allows the algorithms to take the performance advantage at the 10th iteration, with a stronger convergence speed. After the 10th iteration, FTDZOA is still able to reduce the fitness value, which confirms that FTDZOA has a very good global optimization search performance. The above analysis confirms that FTDZOA has a faster convergence speed and convergence accuracy in solving the medium-dimensional FS problem, and tt has higher practicability and reliability.

As can be seen from Figure 14, as the dimension of the FS problem reaches several hundred, all algorithms are effective in reducing the fitness value when solving the high-dimensional FS problem, although all algorithms suffer a loss in convergence speed. In most cases, FTDZOA occupies a clear convergence advantage after the 40th iteration, and the curve is still trending downward after the 40th iteration, which indicates that FTDZOA can continue to be stable in the extraction of effective features. This is due to the fact that the introduction of the fractional order search strategy, triple mean point guidance strategy, and differential strategy in this paper improves the algorithm’s global optimization ability. Through the above analysis, it is confirmed that FTDZOA also has faster convergence speed and convergence accuracy when solving the high-dimensional FS problem, and has higher practicality and reliability.

### 4.8. Feature Subset and Accuracy Analysis

The purpose of FS is mainly to remove the redundant features from the original dataset so as to improve the classification accuracy. Therefore, this subsection mainly analyzes the relationship between feature subset size and classification accuracy during the experiment. Table 7 shows the classification accuracy of the algorithm in solving the FS problem, and Table 8 shows the feature subset size of the algorithm in solving the FS problem, where “Mean Rank” denotes the average rank on all FS problems.

Combining Table 7 and Table 8, it can be seen that when solving the low-dimensional FS problem, all the algorithms can effectively downscale the redundant features of the original dataset and thus improve the classification accuracy, but it is worth noting that FTDZOA achieved excellent feature downsizing performance by having the first accuracy rate on 88% of the low-dimensional FS problem, but it is undeniably weaker than GWO in the Breastcancer dataset, which also reflects that there is still room for improvement of FTDZOA in some specific datasets. Meanwhile, through Table 8, we can find that the feature subset size obtained by FTDZOA is not dominant, which is mainly due to the fact that other algorithms eliminate the useful feature information, thus obtaining a smaller feature subset size, but the classification accuracy is greatly reduced. This also proves that the FS method based on FTDZOA has better global optimization ability, which can effectively jump out of the local optimal feature subset and improve the classification accuracy. When solving the medium-dimensional FS problem, FTDZOA has an accuracy rate of 63% in the medium-dimensional FS problems and the first overall ranking; although the winning rate is reduced, it still has a good solving advantage compared with other algorithms. It can be noticed that FTDZOA is not as effective as other algorithms in Zoo, WDBC, and BreastEW datasets, which indicates that FTDZOA still has the problem of falling into the locally optimal subset of features when solving certain FS problems, which is due to the increase in the dimension of the FS problem, resulting in the complexity of the search space. However, FTDZOA still has the best performance in the medium-dimension FS problem compared with the other algorithms. Compared with the other algorithms, FTDZOA still occupies a certain performance advantage in the middle-dimensional FS problem. When solving the high-dimensional FS problem, it can be seen that the solution performance of the other algorithms is greatly affected due to the sharp increase in the problem dimension, while FTDZOA still achieves excellent results by winning first place with a probability of 72%. It is weaker than DE and EO only in the Musk and Clean datasets. However, it is undeniable that from a comprehensive point of view, FTDZOA occupies a great advantage in solving high-dimensional FS problems. In summary, although FTDZOA does not perform as well as other algorithms in a few specific FS problems, from a comprehensive point of view, FTDZOA can be regarded as a FS method with efficient solution performance, good global optimization performance, and effective removal of redundant features from the dataset.

### 4.9. Runtime Analysis

In the above subsections, the solution performance and reliability of FTDZOA have been verified, but in addition, the actual running time of the algorithm should also be taken into account, which determines the practicality of the algorithm in real-world environments. Therefore, in this section, the running time of the algorithm is mainly analyzed to confirm the practicality of FTDZOA in a real environment. Table 9 shows the running time of the algorithm in solving the FS problem, where “Mean Rank” indicates the average rank of the algorithm in different FS problems. From Table 9, it can be seen that FTDZOA has the least running time on 19 datasets, occupying 83%, which is mainly due to the simplicity of the logic of the FTDZOA algorithm, which means the actual execution time of the algorithm will be greatly saved. Meanwhile, the average ranking shows that FTDZOA has an average ranking of 1.26, which is ahead of the other algorithms. From this, we can conclude that the FS method based on FTDZOA is a highly practical FS method.

### 4.10. Synthesized Analysis

**Figure 15:**
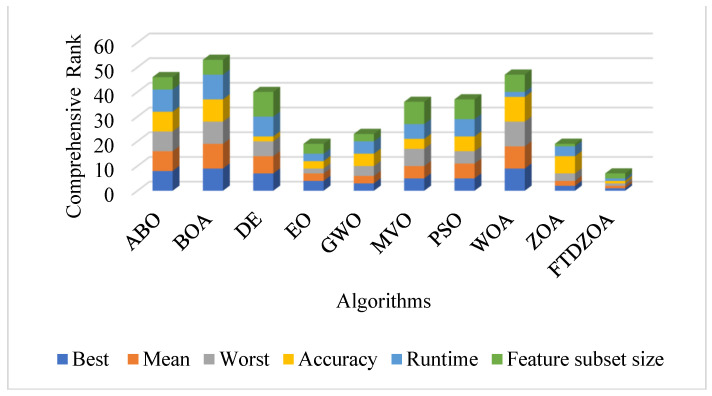
Comprehensive performance stacking chart.

The reliability, stability, and practicality of the FS method based on FTDZOA have been verified in the previous subsections, and in this subsection, the main focus is on the comprehensive analysis of the different evaluation metrics, which involve the best fitness value, the mean fitness value, the worst fitness value, the classification accuracy, the feature subset size, and the running time. Figure 15 shows the bar stacked plot of the average ranking on different metrics, where the lower the height of the bar indicates that the algorithm’s comprehensive performance on the FS problem is better. From Figure 15, it can be seen that FTDZOA has the smallest stacking height, so from a comprehensive point of view, the FS method based on FTDZOA is a FS method with efficient solving performance, robustness, reliability, and practicality.

## 5. Conclusions

In this paper, we address the problem of the original ZOA’s tendency to fall into a subset of locally optimal features when solving the FS problem, resulting in poor classification accuracy. We propose an improved version of ZOA called FTDZOA. Firstly, the fractional order search strategy is introduced to enhance the exploitation ability of ZOA in solving the FS problem, which makes full use of the retention ability of the fractional order search strategy on the information of the previous generation of individuals and improves the classification accuracy. Secondly, the triple mean point guidance strategy is introduced, which combines the information of the global optimal point, random point, and current point to effectively enhance the exploration ability of ZOA, and also ensures the exploitation ability of the algorithm so that the convergence performance of the algorithm is effectively enhanced. Finally, the exploration ability of ZOA is enhanced by introducing the differential strategy, which combines the information difference between different individuals and reduces the risk of falling into the subset of local optimal features. Subsequently, the proposed FTDZOA-based FS method is used to solve 23 FS problems involving low, medium, and high dimensions, and the experimental results confirm that the FTDZOA-based FS method is an efficient, robust, reliable, and practical FS method.

However, it is undeniable that the performance of FTDZOA in certain FS issues still needs improvement. Therefore, in future work, we will propose more effective search strategies tailored to specific feature selection datasets to address this limitation. Additionally, the FTDZOA algorithm presented in this paper is a binary algorithm suitable for solving combinatorial optimization problems. In this paper, only its performance in solving feature selection problems has been evaluated. In future work, we will extend FTDZOA to the field of aviation scheduling, image processing, and natural language processing to tackle challenging problems in real-world scenarios. Furthermore, we will model feature selection issues in real-world scenarios according to the actual situation and develop efficient algorithms for solving specific FS models based on FTDZOA.

## Figures and Tables

**Table 1:** Feature selection algorithm summarization table.

Algorithms	Accuracy	Time	Error	Feature
BFLGWO	Yes	No	Yes	Yes
A-HMDE	Yes	No	Yes	No
ISPSO	No	No	Yes	Yes
LCBCSA	No	Yes	No	Yes
BEGJO	Yes	No	Yes	No
ACD-GOA	No	Yes	No	No
EPO-BFO	No	Yes	No	Yes

**Table 2:** The information on FS datasets.

Category	Name	Features Size	Classification Size	Dataset Size
	Aggregation	2	7	788
	Banana	2	2	5300
	Iris	4	3	150
Low	Bupa	6	2	345
	Glass	9	7	214
	Breastcancer	9	2	699
	Lipid	10	2	583
	HeartEW	13	2	270
	Zoo	16	7	101
	Vote	16	2	435
	Congress	16	2	435
Medium	Lymphography	18	4	148
	Vehicle	18	4	846
	WDBC	30	2	569
	BreastEW	30	2	569
	SonarEW	60	2	208
	Libras	90	15	360
	Hillvalley	100	2	606
	MUSK	166	2	476
High	Clean	167	2	476
	Semeion	256	10	1593
	Meadelon	500	2	2600
	Isolet	617	26	1559

**Table 3:** Comparison algorithm parameter information.

Algorithms	Proposed Time	Parameters Settings
Particle Swarm Optimization (PSO) [43]	1995	$w=1,\;w_{p}=0.99,\;c_{1}=1.5,\;c_{2}=2.0$
Differential Evolution (DE) [44]	1997	F=0.5, CR = 0.9
Grey Wolf Optimizer (GWO) [45]	2014	α=2−2·(FEs−MaxFEs)
Multi-Verse Optimizer (MVO) [46]	2016	$WEP_{Max}=1,\;WEP_{Min}=0.2$
Whale Optimization Algorithm (WOA) [47]	2016	$b=1,\;a_{1}=2-(2\cdot FEs/MaxFEs),\;a_{2}=-1-(FEs/MaxFEs)$
Artificial Butterfly Optimization (ABO) [48]	2017	$ratio_{e}=0.2,\;step_{e}=0.05$
Butterfly Optimization Algorithm (BOA) [49]	2019	p=0.8, α = 0.1, c = 0.01
Equilibrium Optimizer (EO) [50]	2020	$V=1,\;a_{1}=2,\;a_{2}=1,\;GP=0.5$
ZOA	2022	*No p*arameters
FTDZOA	NA	*No p*arameters

**Table 4:** Fitness values on 23 FS datasets.

Category	Datasets	Metric	ABO	BOA	DE	EO	GWO	MVO	PSO	WOA	ZOA	FTDZOA
	Aggregation	Best	**0.100**	0.106	**0.100**	**0.100**	**0.100**	**0.100**	**0.100**	**0.100**	**0.100**	**0.100**
		Mean	**0.100**	0.115	**0.100**	**0.100**	**0.100**	**0.100**	**0.100**	**0.100**	**0.100**	**0.100**
		Worst	**0.100**	0.377	**0.100**	**0.100**	**0.100**	**0.100**	**0.100**	**0.100**	**0.100**	**0.100**
		Rank	1/1/1	0/10/10	1/1/1	1/1/1	1/1/1	1/1/1	1/1/1	1/1/1	1/1/1	1/1/1
	Banana	Best	0.198	0.193	0.191	0.193	0.192	0.203	0.193	0.210	0.198	**0.187**
		Mean	0.198	0.198	0.191	0.193	0.192	0.203	0.193	0.210	0.208	**0.187**
		Worst	0.198	0.365	0.191	0.193	0.192	0.203	0.193	0.210	0.335	**0.187**
		Rank	7/6/6	4/7/10	2/2/2	5/4/4	3/3/3	9/8/7	5/4/4	10/10/8	8/9/9	1/1/1
	Iris	Best	**0.025**	**0.025**	0.080	**0.025**	0.025	0.050	0.055	0.080	**0.025**	**0.025**
		Mean	**0.025**	**0.025**	0.080	**0.025**	0.026	0.051	0.059	0.082	**0.025**	**0.025**
		Worst	**0.025**	**0.025**	0.080	**0.025**	0.050	0.080	0.080	0.110	**0.025**	**0.025**
Low		Rank	1/1/1	1/1/1	9/9/7	1/1/1	1/6/6	7/7/7	8/8/7	9/10/10	1/1/1	1/1/1
	Bupa	Best	0.307	0.367	0.307	0.311	0.298	0.314	0.311	0.311	0.311	**0.251**
		Mean	0.310	0.382	0.334	0.322	0.305	0.323	0.324	0.318	0.318	**0.251**
		Worst	0.350	0.419	0.348	0.341	0.359	0.369	0.389	0.350	0.346	**0.251**
		Rank	3/3/5	0/10/10	3/9/4	5/6/2	2/2/7	9/7/8	5/8/9	5/4/5	5/5/3	1/1/1
	Glass	Best	0.344	0.248	0.279	0.279	0.312	0.226	0.269	0.217	0.280	**0.183**
		Mean	0.352	0.293	0.299	0.284	0.314	0.243	0.279	0.237	0.297	**0.196**
		Worst	0.376	0.322	0.355	0.290	0.323	0.302	0.302	0.271	0.344	**0.248**
		Rank	0/10/10	4/6/6	6/8/9	6/5/3	9/9/7	3/3/5	5/4/4	2/2/2	8/7/8	1/1/1
	Breastcancer	Best	0.059	0.042	0.059	0.053	**0.040**	0.053	0.053	0.059	0.042	0.042
		Mean	0.063	0.052	0.061	0.054	0.043	0.057	0.058	0.066	0.048	**0.043**
		Worst	0.072	0.066	0.077	0.072	0.066	0.070	0.066	0.096	0.066	**0.053**
		Rank	8/9/7	2/4/2	8/8/9	5/5/7	1/2/2	5/6/6	5/7/2	8/10/10	2/3/2	2/1/1
	Lipid	Best	0.258	0.227	0.219	0.263	0.239	0.232	0.232	0.227	0.258	**0.216**
		Mean	0.262	0.243	0.257	0.264	0.247	0.238	0.245	0.253	0.260	**0.233**
		Worst	**0.266**	**0.266**	0.304	**0.266**	0.274	**0.266**	**0.266**	0.297	**0.266**	**0.266**
		Rank	8/9/1	3/3/1	2/7/10	10/10/1	7/5/8	5/2/1	5/4/1	3/6/9	8/8/1	1/1/1
	HeartEW	Best	0.106	0.097	0.105	0.164	0.147	0.147	0.155	0.164	0.137	**0.081**
		Mean	0.136	0.156	0.130	0.182	0.164	0.168	0.169	0.198	0.171	**0.094**
		Worst	0.206	0.208	0.186	0.206	0.238	0.223	0.238	0.246	0.215	**0.165**
		Rank	4/3/3	2/4/5	3/2/2	9/9/3	6/5/8	6/6/7	8/7/8	9/10/10	5/8/6	1/1/1
	Zoo	Best	0.038	0.038	0.044	0.044	0.044	0.031	0.044	0.031	0.031	**0.025**
		Mean	0.103	0.086	0.056	0.066	0.100	0.039	0.081	0.096	0.045	**0.038**
		Worst	0.154	0.121	0.089	0.115	0.154	**0.050**	0.140	0.134	0.089	0.076
		Rank	5/10/9	5/7/6	7/4/3	7/5/5	7/9/9	2/2/1	7/6/8	2/8/7	2/3/3	1/1/2
	Vote	Best	0.035	0.033	0.039	0.035	0.046	0.039	0.039	0.039	**0.006**	**0.006**
		Mean	0.037	0.046	0.054	0.042	0.051	0.055	0.051	0.049	**0.006**	**0.006**
		Worst	0.037	0.066	0.077	0.064	0.058	0.079	0.073	0.079	**0.006**	**0.006**
		Rank	4/3/3	3/5/6	6/9/8	4/4/5	10/8/4	6/10/9	6/7/7	6/6/9	1/1/1	1/1/1
	Congress	Best	0.029	0.033	0.027	0.058	0.037	0.038	0.035	0.056	0.048	**0.006**
		Mean	0.036	0.048	0.036	0.066	0.037	0.049	0.051	0.069	0.048	**0.006**
		Worst	0.037	0.085	0.054	0.068	0.037	0.075	0.073	0.093	0.048	**0.006**
		Rank	3/3/2	4/6/9	2/2/5	10/9/6	6/4/2	7/7/8	5/8/7	9/10/10	8/5/4	1/1/1
Medium	Lymphography	Best	0.095	0.090	0.090	0.115	0.095	0.084	0.090	0.112	0.084	**0.064**
		Mean	0.148	0.154	0.129	0.133	0.154	0.118	0.112	0.140	0.140	**0.078**
		Worst	0.192	0.183	0.200	0.177	0.214	0.177	0.138	0.205	0.172	**0.101**
		Rank	7/8/7	4/10/6	4/4/8	10/5/4	7/9/10	2/3/4	4/2/2	9/6/9	2/7/3	1/1/1
	Vehicle	Best	0.257	0.294	0.268	0.252	0.235	0.237	0.247	0.257	0.225	**0.220**
		Mean	0.291	0.321	0.287	0.275	0.261	0.269	0.278	0.306	0.270	**0.231**
		Worst	0.326	0.370	0.316	0.320	0.279	0.305	0.310	0.343	0.299	**0.257**
		Rank	7/8/8	10/10/10	9/7/6	6/5/7	3/2/2	4/3/4	5/6/5	7/9/9	2/4/3	1/1/1
	WDBC	Best	0.034	0.041	0.041	0.045	**0.026**	0.056	0.041	0.042	0.039	**0.026**
		Mean	0.054	0.064	0.050	0.049	0.052	0.072	0.056	0.063	0.046	**0.033**
		Worst	0.076	0.086	0.064	0.070	0.066	0.090	0.090	0.085	0.062	**0.039**
		Rank	3/6/6	5/9/8	5/4/3	9/3/5	1/5/4	10/10/9	5/7/9	8/8/7	4/2/2	1/1/1
	BreastEW	Best	0.035	0.059	0.040	0.031	0.036	0.027	0.027	0.063	0.031	**0.013**
		Mean	0.045	0.069	0.056	0.042	0.048	0.047	0.041	0.081	0.051	**0.030**
		Worst	0.059	0.080	0.068	0.054	0.065	0.066	0.059	0.098	0.068	**0.041**
		Rank	6/4/3	9/9/9	8/8/8	4/3/2	7/6/5	2/5/6	2/2/3	0/10/10	4/7/7	1/1/1
	SonarEW	Best	0.108	0.069	0.069	**0.013**	0.037	0.054	0.030	0.066	0.032	**0.013**
		Mean	0.139	0.117	0.090	0.033	0.075	0.093	0.048	0.119	0.071	**0.029**
		Worst	0.174	0.170	0.102	0.057	0.106	0.133	0.084	0.182	0.098	**0.049**
		Rank	10/10/9	9/8/8	8/6/5	1/2/2	5/5/6	6/7/7	3/3/3	7/9/10	4/4/4	1/1/1
	Libras	Best	0.116	0.176	0.142	0.130	0.131	0.148	0.172	0.162	0.100	**0.069**
		Mean	0.175	0.201	0.154	0.165	0.150	0.188	0.199	0.214	0.141	**0.106**
		Worst	0.205	0.231	0.164	0.185	0.168	0.217	0.225	0.284	0.176	**0.133**
		Rank	3/6/6	10/9/9	6/4/2	4/5/5	5/3/3	7/7/7	9/8/8	8/10/10	2/2/4	1/1/1
	Hillvalley	Best	0.350	0.334	0.348	0.286	0.265	0.375	0.315	0.300	0.272	**0.256**
		Mean	0.371	0.361	0.371	0.299	0.300	0.408	0.346	0.333	0.296	**0.281**
		Worst	0.394	0.381	0.385	0.321	0.319	0.437	0.369	0.365	0.323	**0.306**
		Rank	9/8/9	7/7/7	8/9/8	4/3/3	2/4/2	0/10/10	6/6/6	5/5/5	3/2/4	1/1/1
	Musk	Best	0.097	0.091	0.072	**0.025**	0.037	0.056	0.070	0.053	0.051	**0.025**
		Mean	0.131	0.114	0.093	0.058	0.063	0.074	0.096	0.081	0.092	**0.054**
		Worst	0.152	0.138	0.110	0.094	0.114	0.115	0.122	0.112	0.120	**0.075**
		Rank	0/10/10	9/9/9	8/7/3	1/2/2	3/3/5	6/4/6	7/8/8	5/5/4	4/6/7	1/1/1
High	Clean	Best	0.081	0.094	0.090	0.020	0.030	0.060	0.067	0.038	0.031	**0.015**
		Mean	0.101	0.111	0.129	0.042	0.062	0.077	0.102	0.085	0.058	**0.039**
		Worst	0.129	0.125	0.161	**0.062**	0.088	0.117	0.162	0.128	0.098	0.074
		Rank	8/7/7	10/9/6	9/10/9	2/2/1	3/4/3	6/5/5	7/8/10	5/6/7	4/3/4	1/1/2
	Semeion	Best	0.114	0.112	0.096	0.090	0.066	0.088	0.102	0.097	0.088	**0.047**
		Mean	0.126	0.126	0.112	0.107	0.085	0.111	0.116	0.118	0.103	**0.064**
		Worst	0.134	0.135	0.123	0.118	0.112	0.130	0.132	0.134	0.112	**0.071**
		Rank	10/9/8	9/10/10	6/6/5	5/4/4	2/2/2	3/5/6	8/7/7	7/8/8	4/3/3	1/1/1
	Meadelon	Best	0.176	0.230	0.217	0.111	0.114	0.183	0.196	0.190	0.113	**0.100**
		Mean	0.260	0.261	0.237	0.162	0.140	0.214	0.225	0.236	0.156	**0.133**
		Worst	0.313	0.304	0.269	0.227	0.193	0.262	0.247	0.271	0.248	**0.181**
		Rank	5/9/10	10/10/9	9/8/7	2/4/3	4/2/2	6/5/6	8/6/4	7/7/8	3/3/5	1/1/1
	Isolet	Best	0.175	0.159	0.180	0.114	0.099	0.152	0.123	0.141	0.106	**0.077**
		Mean	0.193	0.179	0.197	0.139	0.119	0.172	0.147	0.169	0.128	**0.097**
		Worst	0.217	0.204	0.206	0.163	0.143	0.198	0.172	0.193	0.157	**0.115**
		Rank	9/9/10	8/8/8	10/10/9	4/4/4	2/2/2	7/7/7	5/5/5	6/6/6	3/3/3	1/1/1
	Mean Rank	Best	6.130	6.435	6.043	5.000	4.217	5.609	5.609	6.435	3.826	**1.043**
		Mean	6.609	7.435	6.261	4.391	4.391	5.652	5.739	7.217	4.217	**1.000**
		Worst	6.174	7.174	5.783	3.478	4.478	5.957	5.565	7.565	3.826	**1.087**
	Final Rank	Best	8	9	7	4	3	5	5	9	2	**1**
		Mean	8	10	7	3	3	5	6	9	2	**1**
		Worst	8	9	6	2	4	7	5	10	3	**1**

**Table 5:** The rank of the Friedman mean test.

Category	Datasets	ABO	BOA	DE	EO	GWO	MVO	PSO	WOA	ZOA	FTDZOA
	Aggregation	5.95	5.98	5.88	6.08	5.98	5.92	6.13	6.10	5.97	1.00
	Banana	6.97	4.20	2.00	5.47	3.00	8.90	5.47	9.90	8.10	1.00
	Iris	3.60	3.55	9.28	3.65	3.52	6.97	8.03	9.47	3.52	3.42
Low	Bupa	3.40	9.97	6.90	5.90	2.93	6.75	6.03	5.77	6.35	1.00
	Glass	9.97	6.62	6.37	5.58	8.42	2.77	4.98	2.43	6.77	1.10
	Breastcancer	8.28	4.70	7.78	4.85	1.98	6.03	6.48	8.70	3.90	2.28
	Lipid	7.97	4.13	5.87	8.32	5.02	3.48	4.23	6.00	7.20	2.78
	HeartEW	3.63	5.22	2.90	8.10	5.80	5.95	6.20	9.37	6.50	1.33
	Zoo	8.05	7.00	4.58	5.35	7.72	2.53	6.87	7.65	3.00	2.25
	Vote	3.72	6.13	7.93	4.93	7.70	7.82	7.23	6.53	1.52	1.48
	Congress	3.37	5.88	3.38	8.97	3.70	6.67	6.37	9.22	6.45	1.00
Medium	Lymphography	7.32	7.83	5.33	5.80	7.53	4.23	3.52	6.10	6.32	1.02
	Vehicle	7.08	9.58	6.63	5.02	2.95	4.28	5.35	8.25	4.68	1.17
	WDBC	5.37	7.77	5.15	4.17	5.63	9.03	5.88	7.28	3.60	1.12
	BreastEW	4.58	8.88	7.28	3.72	5.07	4.80	3.60	9.88	5.62	1.57
	SonarEW	9.43	8.22	6.17	2.07	5.03	6.60	2.98	8.30	4.62	1.58
	Libras	6.03	8.33	3.77	5.07	3.52	7.40	8.37	8.93	2.55	1.03
	Hillvalley	7.97	7.30	8.28	2.93	3.12	9.97	6.08	5.33	2.57	1.45
High	Musk	9.70	8.65	6.63	2.53	2.90	4.17	6.87	5.02	6.50	2.03
	Clean	7.35	8.67	9.60	2.02	3.75	5.18	7.47	5.93	3.33	1.70
	Semeion	9.07	9.13	5.77	4.70	2.10	5.55	6.62	7.47	3.57	1.03
	Madelon	8.77	9.10	7.50	3.20	2.37	5.47	6.47	7.27	2.93	1.93
	Isolet	9.10	7.73	9.37	3.93	2.57	6.83	4.53	6.63	3.27	1.03
	Mean Rank	6.81	7.16	6.28	4.88	4.45	5.97	5.90	7.28	4.73	1.54
	Final Rank	8	9	7	4	2	6	5	10	3	1

**Table 6:** Wilcoxon statistical rank sum test results.

Category	Datasets	ABO	BOA	DE	EO	GWO	MVO	PSO	WOA	ZOA
	Aggregation	4.16 × 10^−14^/−	4.16 × 10^−14^/−	6.14 × 10^−14^/−	6.14 × 10^−14^/−	4.16 × 10^−14^/−	4.16 × 10^−14^/−	6.14 × 10^−14^/−	6.14 × 10^−14^/−	4.16 × 10^−14^/−
	Banana	1.69 × 10^−14^/−	2.71 × 10^−14^/−	1.69 × 10^−14^/−	1.69 × 10^−14^/−	1.69 × 10^−14^/−	1.69 × 10^−14^/−	1.69 × 10^−14^/−	1.69 × 10^−14^/−	4.16 × 10^−14^/−
	Iris	3.34 × 10^−1^/=	3.34 × 10^−1^/=	1.69 × 10^−14^/−	3.34 × 10^−1^/=	3.34 × 10^−1^/=	4.16 × 10^−14^/−	1.18 × 10^−13^/−	1.19 × 10^−13^/−	3.34 × 10^−1^/=
Low	Bupa	4.16 × 10^−14^/−	9.27 × 10^−13^/−	7.56 × 10^−13^/−	3.80 × 10^−13^/−	8.70 × 10^−14^/−	4.98 × 10^−13^/−	3.00 × 10^−13^/−	6.49 × 10^−13^/−	6.89 × 10^−13^/−
	Glass	7.21 × 10^−12^/−	1.01 × 10^−11^/−	7.71 × 10^−12^/−	6.81 × 10^−12^/−	6.42 × 10^−12^/−	2.57 × 10^−9^/−	7.27 × 10^−12^/−	9.75 × 10^−9^/−	8.14 × 10^−12^/−
	Breastcancer	4.92 × 10^−12^/−	1.50 × 10^−7^/−	3.35 × 10^−12^/−	1.76 × 10^−11^/−	1.16 × 10^−4^/−	1.42 × 10^−11^/−	1.68 × 10^−11^/−	6.31 × 10^−12^/−	7.35 × 10^−2^/=
	Lipid	1.13 × 10^−10^/−	8.62 × 10^−3^/−	1.04 × 10^−3^/−	1.23 × 10^−10^/−	8.49 × 10^−4^/−	5.65 × 10^−2^/=	3.49 × 10^−3^/−	1.39 × 10^−5^/−	1.27 × 10^−10^/−
	HeartEW	4.42 × 10^−8^/−	4.14 × 10^−10^/−	2.99 × 10^−7^/−	2.60 × 10^−11^/−	8.80 × 10^−11^/−	7.66 × 10^−11^/−	6.74 × 10^−11^/−	1.14 × 10^−11^/−	5.22 × 10^−11^/−
	Zoo	1.78 × 10^−9^/−	1.35 × 10^−9^/−	1.53 × 10^−7^/−	1.13 × 10^−8^/−	1.86 × 10^−10^/−	1.08 × 10^−1^/=	1.11 × 10^−9^/−	1.23 × 10^−9^/−	2.26 × 10^−2^/−
	Vote	1.97 × 10^−13^/−	1.13 × 10^−12^/−	1.15 × 10^−12^/−	6.52 × 10^−13^/−	7.82 × 10^−13^/−	1.18 × 10^−12^/−	1.08 × 10^−12^/−	1.59 × 10^−13^/−	3.34 × 10^−1^/=
	Congress	1.58 × 10^−13^/−	1.06 × 10^−12^/−	1.03 × 10^−12^/−	3.77 × 10^−13^/−	1.69 × 10^−14^/−	1.16 × 10^−12^/−	1.17 × 10^−12^/−	8.79 × 10^−13^/−	1.69 × 10^−14^/−
Medium	Lymphography	4.99 × 10^−11^/−	5.00 × 10^−11^/−	2.41 × 10^−10^/−	2.62 × 10^−11^/−	3.12 × 10^−11^/−	3.42 × 10^−9^/−	1.41 × 10^−10^/−	2.34 × 10^−11^/−	1.58 × 10^−10^/−
	Vehicle	2.79 × 10^−11^/−	2.65 × 10^−11^/−	2.62 × 10^−11^/−	3.78 × 10^−11^/−	1.30 × 10^−9^/−	5.20 × 10^−10^/−	8.80 × 10^−11^/−	2.81 × 10^−11^/−	1.91 × 10^−9^/−
	WDBC	5.00 × 10^−10^/−	1.92 × 10^−11^/−	1.69 × 10^−11^/−	1.03 × 10^−11^/−	2.72 × 10^−10^/−	1.89 × 10^−11^/−	1.87 × 10^−11^/−	1.90 × 10^−11^/−	2.74 × 10^−9^/−
	BreastEW	1.06 × 10^−9^/−	2.90 × 10^−11^/−	3.89 × 10^−11^/−	1.78 × 10^−8^/−	8.80 × 10^−11^/−	2.98 × 10^−8^/−	9.41 × 10^−6^/−	2.92 × 10^−11^/−	4.39 × 10^−10^/−
	SonarEW	2.91 × 10^−11^/−	2.92 × 10^−11^/−	2.77 × 10^−11^/−	9.98 × 10^−2^/=	2.06 × 10^−10^/−	2.91 × 10^−11^/−	2.90 × 10^−5^/−	2.93 × 10^−11^/−	2.38 × 10^−10^/−
	Libras	6.96 × 10^−11^/−	2.98 × 10^−11^/−	2.92 × 10^−11^/−	4.02 × 10^−11^/−	3.29 × 10^−11^/−	2.97 × 10^−11^/−	2.97 × 10^−11^/−	2.98 × 10^−11^/−	2.20 × 10^−9^/−
	Hillvalley	2.90 × 10^−11^/−	3.01 × 10^−11^/−	3.00 × 10^−11^/−	6.74 × 10^−8^/−	1.35 × 10^−7^/−	3.00 × 10^−11^/−	3.01 × 10^−11^/−	3.29 × 10^−11^/−	6.75 × 10^−5^/−
High	Musk	3.01 × 10^−11^/−	3.01 × 10^−11^/−	4.06 × 10^−11^/−	2.74 × 10^−1^/=	7.24 × 10^−2^/=	9.18 × 10^−6^/−	7.37 × 10^−11^/−	1.05 × 10^−8^/−	6.70 × 10^−10^/−
	Clean	3.00 × 10^−11^/−	3.01 × 10^−11^/−	3.01 × 10^−11^/−	2.67 × 10^−1^/=	3.37 × 10^−7^/−	1.32 × 10^−10^/−	4.06 × 10^−11^/−	6.11 × 10^−10^/−	3.32 × 10^−6^/−
	Semeion	3.01 × 10^−11^/−	3.01 × 10^−11^/−	3.02 × 10^−11^/−	3.01 × 10^−11^/−	9.42 × 10^−11^/−	3.02 × 10^−11^/−	3.02 × 10^−11^/−	3.02 × 10^−11^/−	3.02 × 10^−11^/−
	Meadelon	3.34 × 10^−11^/−	3.02 × 10^−11^/−	3.02 × 10^−11^/−	4.71 × 10^−4^/−	1.76 × 10^−1^/=	3.02 × 10^−11^/−	3.02 × 10^−11^/−	3.02 × 10^−11^/−	3.03 × 10^−3^/−
	Isolet	3.02 × 10^−11^/−	3.02 × 10^−11^/−	3.02 × 10^−11^/−	3.34 × 10^−11^/−	1.56 × 10^−8^/−	3.02 × 10^−11^/−	3.01 × 10^−11^/−	3.02 × 10^−11^/−	1.46 × 10^−10^/−
	+/−/=	0/22/1	0/22/1	0/23/0	0/19/4	0/20/3	0/21/2	0/23/0	0/23/0	0/20/3

**Table 7:** Classification accuracy on the FS problem.

Datasets	ABO	BOA	DE	EO	GWO	MVO	PSO	WOA	ZOA	FTDZOA
Aggregation	100.00	96.38	100.00	100.00	100.00	100.00	100.00	100.00	100.00	**100.00**
	1	10	1	1	1	1	1	1	1	**1**
Banana	89.15	88.89	89.91	89.62	89.81	88.58	89.62	87.83	87.67	**90.38**
	6	7	2	4	3	8	4	9	10	**1**
Iris	100.00	100.00	96.67	100.00	100.00	99.78	96.67	96.11	100.00	**100.00**
	1	1	8	1	1	7	8	10	1	**1**
Bupa	69.37	63.14	70.43	70.48	71.59	70.58	69.57	69.13	69.37	**73.91**
	8	10	5	4	2	3	6	9	7	**1**
Glass	63.10	71.90	70.16	72.46	70.00	76.90	73.57	80.00	71.67	**82.06**
	10	6	8	5	9	3	4	2	7	**1**
Breastcancer	96.35	96.79	96.86	97.29	**98.47**	97.22	97.10	95.85	97.29	97.60
	9	8	7	3	**1**	5	6	10	3	2
Lipid	71.95	74.68	74.14	73.36	75.26	76.64	75.95	73.13	72.47	**76.67**
	10	5	6	7	4	2	3	8	9	**1**
HeartEW	88.58	85.86	90.06	82.59	85.19	85.31	85.62	80.12	83.58	**93.02**
	3	4	2	9	7	6	5	10	8	**1**
Zoo	92.17	95.33	98.50	96.83	92.67	**100.00**	96.00	94.83	99.33	99.67
	10	7	4	5	9	**1**	6	8	3	2
Vote	97.09	97.70	96.93	98.08	96.17	96.82	96.67	95.71	100.00	**100.00**
	5	4	6	3	9	7	8	10	1	**1**
Congress	97.01	97.43	98.81	93.95	96.55	97.43	97.24	94.33	95.40	**100.00**
	6	3	2	10	7	4	5	9	8	**1**
Lymphography	87.36	86.32	90.11	88.74	85.52	90.92	92.07	87.24	86.78	**95.40**
	6	9	4	5	10	3	2	7	8	**1**
Vehicle	71.44	67.97	72.90	73.12	74.42	74.75	73.63	69.70	73.61	**77.44**
	8	10	7	6	3	2	4	9	5	**1**
WDBC	95.87	95.46	**97.35**	95.55	95.37	94.72	96.28	94.48	95.87	97.17
	4	7	**1**	6	8	9	3	10	5	2
BreastEW	98.14	96.28	98.85	97.94	97.26	**98.91**	98.82	94.37	96.43	98.64
	5	9	2	6	7	**1**	3	10	8	4
SonarEW	87.48	89.67	95.85	98.37	93.58	94.23	98.37	89.92	93.98	**98.70**
	10	9	4	2	7	5	2	8	6	**1**
Libras	83.33	80.00	88.89	83.70	85.60	84.03	82.18	78.43	86.16	**90.79**
	7	9	2	6	4	5	8	10	3	**1**
Hillvalley	60.17	61.35	65.70	68.68	67.47	59.45	66.23	63.58	67.99	**71.46**
	9	8	6	2	4	10	5	7	3	**1**
Musk	89.37	90.84	**96.74**	95.96	95.89	96.60	94.21	95.30	91.58	96.70
	10	9	**1**	4	5	3	7	6	8	2
Clean	92.00	90.88	92.84	**98.07**	95.96	96.39	93.37	94.88	95.93	97.93
	9	10	8	**1**	4	3	7	6	5	2
Semeion	91.25	90.25	94.79	93.38	94.15	93.11	92.47	91.99	93.87	**96.72**
	9	10	2	5	3	6	7	8	4	**1**
Madelon	75.67	74.07	81.18	84.08	86.80	81.72	80.38	77.75	83.90	**87.11**
	9	10	6	3	2	5	7	8	4	**1**
Isolet	82.08	82.68	85.36	87.32	89.57	86.29	88.95	84.48	88.40	**91.21**
	10	9	7	5	2	6	3	8	4	**1**
Mean Rank	7.17	7.57	4.39	4.48	4.87	4.57	4.96	7.96	5.26	**1.35**
Final Rank	8	9	2	3	5	4	6	10	7	**1**

**Table 8:** Feature subset size on the FS problem.

Datasets	ABO	BOA	DE	EO	GWO	MVO	PSO	WOA	ZOA	FTDZOA
Aggregation	2.00	**1.97**	2.00	2.00	2.00	2.00	2.00	2.00	2.00	2.00
	2	**1**	2	2	2	2	2	2	2	2
Banana	2.00	1.97	2.00	2.00	2.00	2.00	2.00	2.00	**1.93**	2.00
	3	2	3	3	3	3	3	3	**1**	3
Iris	1.00	1.00	2.00	1.00	1.03	1.97	1.17	1.87	1.00	**1.00**
	1	1	10	1	6	9	7	8	1	**1**
Bupa	2.07	3.03	4.07	3.37	2.97	3.47	3.00	2.43	2.57	**1.00**
	2	7	10	8	5	9	6	3	4	**1**
Glass	**1.80**	3.60	2.70	3.27	3.93	3.20	3.67	5.10	3.77	3.13
	**1**	6	2	5	9	4	7	10	8	3
Breastcancer	2.70	2.07	2.97	2.70	2.60	2.87	2.90	2.60	2.10	**1.90**
	6	2	10	6	4	8	9	4	3	**1**
Lipid	**1.00**	1.50	2.47	2.40	2.47	2.80	2.83	1.17	1.20	2.33
	**1**	4	7	6	7	9	10	2	3	5
HeartEW	4.33	3.80	5.23	3.30	4.00	4.63	5.20	**2.47**	2.97	4.00
	7	4	10	3	5	8	9	**1**	2	5
Zoo	**5.17**	7.00	6.73	6.07	5.50	6.30	7.17	7.97	6.20	5.67
	**1**	8	7	4	2	6	9	10	5	3
Vote	1.70	4.00	4.27	3.97	2.67	4.27	3.30	1.60	1.00	**1.00**
	4	8	9	7	5	9	6	3	1	**1**
Congress	1.53	3.93	4.10	1.87	1.00	4.17	4.17	2.87	1.00	**1.00**
	4	7	8	5	1	9	9	6	1	**1**
Lymphography	6.23	5.63	7.23	5.70	4.30	6.50	7.33	4.50	**3.83**	6.63
	6	4	9	5	2	7	10	3	**1**	8
Vehicle	6.20	5.93	7.80	5.97	5.50	7.50	7.27	5.93	5.80	**5.00**
	7	4	10	6	2	9	8	4	3	**1**
WDBC	4.93	7.00	7.87	2.67	3.07	7.23	6.90	3.87	2.60	**2.17**
	6	8	10	3	4	9	7	5	2	**1**
BreastEW	8.57	10.77	13.80	7.03	7.00	11.10	9.17	9.17	5.73	**5.20**
	5	8	10	4	3	9	6	6	2	**1**
SonarEW	15.67	14.50	31.60	11.23	10.20	24.53	20.23	17.17	**10.13**	10.27
	6	5	10	4	2	9	8	7	**1**	3
Libras	22.37	19.03	48.70	16.50	18.80	39.60	35.17	18.17	**14.67**	20.63
	7	5	10	2	4	9	8	3	**1**	6
Hillvalley	12.13	13.10	62.67	17.03	7.50	42.83	41.67	**5.63**	7.93	24.30
	4	5	10	6	2	9	8	**1**	3	7
Musk	57.83	51.87	105.13	36.57	42.87	72.20	73.27	64.03	**26.50**	40.03
	6	5	10	2	4	8	9	7	**1**	3
Clean	48.53	47.83	107.03	41.13	42.67	74.00	70.43	64.73	36.07	**34.57**
	6	5	10	3	4	9	8	7	2	**1**
Semeion	119.83	98.20	167.70	121.57	**83.37**	125.67	122.93	118.23	122.07	87.93
	5	3	10	6	**1**	9	8	4	7	2
Madelon	205.67	140.60	337.17	92.03	108.23	245.93	243.73	178.10	**55.10**	86.20
	7	5	10	3	4	9	8	6	**1**	2
Isolet	198.00	145.40	401.17	152.93	154.87	297.43	295.90	181.63	146.83	**112.37**
	7	2	10	4	5	9	8	6	3	**1**
Mean Rank	4.52	4.74	8.565217	4.26	3.74	7.87	7.52	4.83	**2.52**	2.70
Final Rank	5	6	10	4	3	9	8	7	**1**	2

**Table 9:** Runtime on the FS problem.

Datasets	ABO	BOA	DE	EO	GWO	MVO	PSO	WOA	ZOA	FTDZOA
Aggregation	5.60	6.11	3.51	2.70	3.39	3.46	3.44	3.25	**2.03**	2.59
	9	10	8	3	5	7	6	4	**1**	2
Banana	10.22	11.17	6.56	4.81	6.10	6.42	6.30	6.10	**3.31**	4.54
	9	10	8	3	5	7	6	4	**1**	2
Iris	4.34	5.01	3.16	2.91	3.09	3.11	2.96	2.90	2.51	**1.95**
	9	10	8	4	6	7	5	3	2	**1**
Bupa	5.27	5.69	3.28	3.22	3.23	3.27	3.26	2.97	2.84	**2.19**
	9	10	8	4	5	7	6	3	2	**1**
Glass	5.39	5.27	3.20	3.17	3.18	3.19	3.19	3.01	2.99	**2.13**
	10	9	8	4	5	6	7	3	2	**1**
Breastcancer	5.13	5.69	3.49	3.41	3.44	3.47	3.48	3.20	3.43	**2.27**
	9	10	8	3	5	6	7	2	4	**1**
Lipid	4.62	5.79	3.42	3.30	3.31	3.37	3.43	2.73	3.25	**2.05**
	9	10	7	4	5	6	8	2	3	**1**
HeartEW	5.16	5.78	3.26	3.21	3.24	3.26	3.26	2.80	3.22	**2.14**
	9	10	8	3	5	7	6	2	4	**1**
Zoo	5.88	5.77	3.13	3.13	3.14	3.17	3.15	2.93	3.10	**2.15**
	10	9	5	4	6	8	7	2	3	**1**
Vote	4.37	5.89	3.34	3.22	3.28	3.34	3.33	2.74	3.31	**2.11**
	9	10	7	3	4	8	6	2	5	**1**
Congress	4.36	5.07	3.32	3.25	3.27	3.34	3.34	2.85	3.30	**2.06**
	9	10	6	3	4	7	8	2	5	**1**
Lymphography	5.14	5.60	3.17	3.18	3.18	3.19	3.20	2.78	3.15	**2.12**
	9	10	4	5	6	7	8	2	3	**1**
Vehicle	6.43	6.69	3.61	3.61	3.61	3.66	3.67	3.34	3.52	**2.48**
	9	10	5	4	6	7	8	2	3	**1**
WDBC	5.12	5.65	3.34	3.39	3.42	3.41	3.44	2.98	3.33	**2.13**
	9	10	4	5	7	6	8	2	3	**1**
BreastEW	6.04	6.24	3.16	3.49	3.49	3.29	3.40	3.14	3.36	**2.23**
	9	10	3	7	8	4	6	2	5	**1**
SonarEW	5.90	5.85	3.05	3.04	3.06	3.05	3.07	2.89	3.04	**2.07**
	10	9	6	4	7	5	8	2	3	**1**
Libras	6.29	6.13	3.29	3.13	3.14	3.25	3.29	3.03	3.18	**2.20**
	10	9	8	3	4	6	7	2	5	**1**
Hillvalley	5.93	6.23	3.68	3.23	3.29	3.49	3.55	3.03	3.35	**2.20**
	9	10	8	3	4	6	7	2	5	**1**
Musk	6.43	6.33	3.78	3.29	3.34	3.58	3.66	3.27	3.39	**2.40**
	10	9	8	3	4	6	7	2	5	**1**
Clean	6.57	6.29	3.80	3.33	3.35	3.56	3.67	3.27	3.40	**2.41**
	10	9	8	3	4	6	7	2	5	**1**
Semeion	17.28	18.53	14.15	8.32	8.13	11.12	11.22	10.19	8.45	**7.10**
	9	10	8	3	2	6	7	5	4	**1**
Madelon	23.76	34.92	58.25	**19.77**	22.83	42.06	42.16	30.88	27.94	24.78
	3	7	10	**1**	2	8	9	6	5	4
Isolet	20.18	23.14	29.85	**11.30**	14.57	22.64	23.07	14.79	16.48	13.36
	6	9	10	**1**	3	7	8	4	5	2
Mean Rank	8.87	9.57	7.09	3.48	4.87	6.52	7.04	2.70	3.61	**1.26**
Final Rank	9	10	8	3	5	6	7	2	4	**1**

## Data Availability

All data in this paper can be obtained by contacting the corresponding author.
